# A cross‐site comparison reveals limited geographic variation in risk avoidance of snowshoe hares

**DOI:** 10.1002/ecy.70378

**Published:** 2026-04-10

**Authors:** Shotaro Shiratsuru, Emily K. Studd, Michael J. L. Peers, Yasmine N. Majchrzak, Alice J. Kenney, Dennis L. Murray, Mark Romanski, Jerrold L. Belant, Hailey M. Boone, Stan Boutin, Jonathan N. Pauli

**Affiliations:** ^1^ Department of Forest and Wildlife Ecology University of Wisconsin Madison Wisconsin USA; ^2^ Department of Biological Sciences Thompson Rivers University Kamloops British Columbia Canada; ^3^ Department of Biology Memorial University St. John's Newfoundland Canada; ^4^ Independent Researcher Yukon Canada; ^5^ Department of Biology Trent University Peterborough Ontario Canada; ^6^ Isle Royale National Park, US National Park Service Houghton Michigan USA; ^7^ Department of Fisheries and Wildlife Michigan State University Lansing Michigan USA; ^8^ Department of Biological Sciences University of Alberta Edmonton Alberta Canada

**Keywords:** antipredator response, diel activity, *Lepus americanus*, movement, predation risk, predator–prey, step selection

## Abstract

Predation risk induces a myriad of behavioral antipredator responses of prey. However, different antipredator behaviors exhibit different degrees of flexibility within a single prey species. Moreover, how predator species composition and traits interact with the flexibility of particular antipredator behaviors to determine spatiotemporal activity patterns of prey remains unknown. We examined geographic variation in multiple behavioral axes of a single prey species, snowshoe hares *Lepus americanus*, across three different ecosystems in northern North America that differed in the predator guild (Wisconsin, USA, with multiple generalist predators; Isle Royale, Michigan, USA, with a single generalist predator; and Yukon, Canada, with a specialist ambush predator and a generalist predator). We hypothesized that predator species composition and traits would drive divergent behavioral patterns of hares across ecosystems. Using a biologging‐based dataset of hare behaviors and a camera‐based dataset of predator occurrence collected over multiple winters, we examined diel activity patterns, space use, and movement of hares while accounting for variable environmental conditions. Hares were only slightly less nocturnal (<10%) when faced with a single nocturnal generalist predator (Isle Royale), and they exhibited highly comparable diel activity patterns across all sites. Patterns of spatial risk avoidance of hares were moderately different between the multi‐predator systems; hares potentially avoided more lethal predators in Wisconsin and exhibited the opposite patterns of habitat use between daytime and nighttime in Yukon, possibly to avoid the most active predator at each time of day. Additionally, hare movements were shorter and more tortuous where ambush specialist predators were present (Yukon). Our study demonstrates that spatiotemporal risk avoidance of prey can exhibit limited geographic variation, but certain behavioral responses, such as diel activity, may be more conserved across ecosystems.

## INTRODUCTION

Predators induce a myriad of proactive antipredator responses in their prey (Creel, 2018; Lima & Dill, 1990). Earlier studies, largely focused on the spatial aspect of risk avoidance, revealed that prey avoid locations with high predation risk (Fortin et al., 2005) or reduce foraging efforts in those risky places (Laundré et al., 2001). More recent studies have assessed temporal risk avoidance and have shown that prey often reduce their movement and activity rates at times of day when predators are active (Cunningham et al., 2019; Kohl et al., 2018). Moreover, spatial risk avoidance of prey tends to be more pronounced during times of day when predators are most active (Smith et al., 2019). These findings highlight that the behavioral patterns of prey are shaped by their efforts to minimize spatiotemporal overlap with their predators (Lima & Dill, 1990). Thus, the intersection between the spatial and temporal aspects of risk avoidance has become an area of active exploration in predator–prey ecology (Guiden et al., 2019).

Predator species composition and traits mediate the effects of risk on prey behaviors (Wirsing et al., 2021). For example, predator traits such as hunting mode can determine the magnitude of behavioral antipredator responses (Preisser et al., 2007). Sit‐and‐wait/pursue (hereafter ambush) predators emit more reliable spatial risk cues compared to actively hunting or cruising (hereafter active) predators, inducing a larger magnitude of spatial risk avoidance of prey (Schmitz, 2007). Ambush predators can also cause prey movement to be shorter and less directional (Avgar et al., 2008). Additionally, the efficacy of spatiotemporal risk avoidance of prey can differ between single‐ and multi‐predator systems. In single‐predator systems, prey can simply avoid high risk locations at times of day when the predator is active (Smith et al., 2019). In contrast, in multi‐predator systems where multiple predators exhibit similar spatiotemporal activity patterns, prey should either prioritize avoiding predators that emit reliable spatial cues (Preisser et al., 2007; Thaker et al., 2011) or more lethal predators (Lima, 1992; Relyea, 2003). Finally, predator traits such as sensory modalities or morphological traits can interact with environmental conditions to further mediate antipredator behaviors. For example, light level can have a larger effect on risk avoidance by prey when their predators rely on visual cues (Kronfeld‐Schor et al., 2013). Overall, a prey species can exhibit divergent patterns of antipredator behaviors across different ecosystems or geographic regions (Large & Smee, 2013) due to differences in predator traits and species composition as well as environmental conditions (Wirsing et al., 2021).

Not all antipredator behaviors are equally flexible (David et al., 2014). The degree of persistence of antipredator behaviors can depend on their underlying mechanisms (e.g., genetic basis vs. phenotypic plasticity; Blumstein & Daniel, 2005) or costs in assessing risk (probability of mortality; Flecker, 1992; Sih, 1987). For example, diel activity patterns or responses to moonlight can be inflexible, provided that the underlying endogenous rhythms appear evolutionarily constrained (Kronfeld‐Schor et al., 2013; Kronfeld‐Schor & Dayan, 2003; Prugh & Golden, 2014). Thus, different types of antipredator behaviors can exhibit different degrees of geographic variation within a single prey species depending on their inherent flexibility, as suggested by previous studies (Endler, 1995; Lahti et al., 2009). This highlights the need to understand how predator species composition and traits interact with the flexibility of particular antipredator behaviors, to characterize the spatiotemporal patterns of prey behaviors.

Snowshoe hares *Lepus americanus* occur in snow‐dominated forests throughout northern North America (Krebs et al., 2018; Wilson et al., 2019). Hares are important prey for diverse suite of predators (Feierabend & Kielland, 2015), and previous studies have reported a variety of antipredator behaviors in hares (Majchrzak et al., 2022; Shiratsuru et al., 2021; Studd et al., 2022). Notably, the predator guilds hares are exposed to vary geographically; northern hare populations are sympatric with the snow‐adapted specialist predator, Canada lynx *Lynx canadensis* (Krebs et al., 2018), whereas their southern populations are mainly exposed to generalist predators such as coyote *Canis latrans* and fisher *Pekania pennanti* (Sievert & Keith, 1985). Therefore, differences in the predator species composition and traits may interact with environmental conditions to drive divergent behavioral patterns of hares among the ecosystems.

Using a biologging‐based dataset of hare behaviors and a camera‐based dataset of predator occurrence collected in three different sites in northern North America, we compared hare behaviors while accounting for variable environmental conditions. Specifically, we focused on diel activity patterns, space use, and movement of hares in relation to risk avoidance. The three sites differed in the predator guild; Wisconsin, USA, with multiple generalists that are primarily canid (coyote and red fox *Vulpes vulpes*) and mustelid (fisher and ermine *Mustela erminea*) predators, Isle Royale National Park, USA, with a single generalist, fox, and Yukon, Canada, with a single ambush specialist, lynx (Murray et al., 1995), and a single generalist, coyote. We hypothesized that predator species composition and traits would drive divergent behavioral patterns of hares among the ecosystems. We predicted that the degree of nocturnality of hares would be lower where the daytime activity level of predators is lower, leading to distinct diel activity patterns among the systems. As for space use, we predicted that hares would avoid habitat features selected by their predators, with stronger avoidance shown in the diel phase of higher predator activity in a single‐predator system (Smith et al., 2019), Isle Royale. For multi‐predator systems, we predicted that hares would prioritize avoidance of predators emitting reliable spatial cues (ambush predators, lynx, in Yukon) or more lethal predators (canids in Wisconsin; Sievert & Keith, 1985, and lynx in Yukon; Peers et al., 2020), if multiple predators are active during the same diel phase(s) (Kohl et al., 2019; Thaker et al., 2011). Alternatively, we also predicted that hares would switch the pattern of space use between daytime and night to prioritize avoiding the most active or risky predator during each diel phase (Kohl et al., 2019). Regarding hare movement, we predicted that the presence of the ambush specialist (lynx) would result in shorter and less directional movement of hares in Yukon (Avgar et al., 2008).

## METHODS

### Study area

We collected hare behavioral data and predator detection data in winter at three different sites across northern North America: (1) Wisconsin (the Chequamegon National Forest Medford District, Wisconsin, USA [45° N, 91° W]), (2) Isle Royale (the western end of Isle Royale National Park, Michigan, USA [48° N, 89° W]), and (3) Yukon (the Kluane region, Yukon, Canada [61° N, 138° W]) (Appendix S1: Figure S1). Primary habitat types are conifer–deciduous mixed forests with understory shrubs in Wisconsin (Ribic et al., 2017), northern hardwood forests, conifer swamps, and conifer–deciduous mixed forests in Isle Royale (Rodriguez Curras et al., 2024), and conifer‐dominant forests with understory shrubs in Yukon (Boonstra et al., 2018, see Appendix S1: Methods S1).

Due to the large latitudinal differences, night length is shorter in Wisconsin and Isle Royale (14.07 and 14.33 h, respectively) compared to Yukon (16.27 h) at the beginning of winter (January 1st). Night length then becomes equal across all study sites in early March (11.5 h on March 8), but then becomes longer in Wisconsin and Isle Royale (8.68 and 8.40 h, respectively) than in Yukon (6.07 h) toward the end of our study period (April 30). Based on the weather station data we used in the analyses, daily mean temperature from January 1st to April 30 was higher in Wisconsin (ranged from −22.8 to 19.4°C with median −2.7°C) and Isle Royale (ranged from −22.5 to 9.6°C with median −4.2°C) compared to Yukon (ranged from −39.0 to 8.7°C with median −9.0°C). Daily snow depth was greater in Wisconsin (ranged from 0.0 to 53.3 cm with median 15.62 cm) and Isle Royale (ranged from 0.0 to 64.9 cm with median 20.7 cm) compared to Yukon (ranged from 0.0 to 31.0 cm with median 11.0 cm).

Predators in Wisconsin are primarily mammalian generalists (coyote, red fox, fisher, and ermine), though avian predators are also present (Sievert & Keith, 1985, see Appendix S1: Methods S1). On Isle Royale, red fox, a generalist, is the only primary predator for hares (Johnson, 1970) and predation by other predators is not common (see Appendix S1: Methods S1). Primary predators of hares in Yukon are lynx, a specialist, and coyote, a generalist, while avian predators are secondary (Boutin et al., 1986, see Appendix S1: Methods S1).

### Data collection

We collected predator detection/nondetection data using trail cameras in two winters (January–April) in each of our study sites (Wisconsin: 2022–2023, Isle Royale: 2021–2022, Yukon: 2018–2019). Cameras were deployed in comparable semi‐random spatial distributions across our study sites (Bushnell Outdoor Products, Cody Overland Park, Kansas, USA for Wisconsin [48 cameras in 2022 and 29 cameras in 2023], Stealth Cam DS4K, Irving, Texas, USA for Isle Royale [40 cameras in 2021 and 37 cameras in 2022], Reconyx Inc., Holmen, Wisconsin, USA for Yukon [74 cameras in 2018 and 68 cameras in 2019], see Appendix S1: Methods S2 for details).

We collected behavioral data from GPS/accelerometer collared (Technosmart Europe Srl., Rome, Italy) hares January–April for multiple winters at each study site (Wisconsin: 2022–2023, Isle Royale: 2022–2024, Yukon: 2018–2019). Hares were live‐captured using Tomahawk live‐traps (Tomahawk Live Trap Co., Tomahawk, Wisconsin, USA), and individual ID on the ear‐tag and sex were recorded upon each capture (see Appendix S1: Methods S2). GPS fix rates were set at 2 or 4 h in Wisconsin and Isle Royale, and at 5, 15, or 30 min in Yukon. We retained GPS data points with Horizontal Dilution of Precision (HDOP) ≤ 5 and with ≥3 satellites contacted. Accelerometers recorded body acceleration along three (dorsoventral, anterior–posterior, and lateral) axes at 1 Hz frequency with ±8 g forces. We classified accelerometer data into not moving, foraging (feeding and travel with one hop), hopping, and sprinting using a hierarchical decision tree for hares developed by Studd et al. (2019). We combined hopping, sprinting, and foraging into a single category, active state, to calculate their activity time and rate. For Yukon, we used classified activity data that were previously published and made publicly accessible (Shiratsuru et al., 2023).

We obtained meteorological data, including wind speed, air temperature, and daily snow depth for each study site (see Appendix S1: Methods S1). For Wisconsin, we used wind speed and temperature data recorded every 20 min and daily snow data from the nearest weather station to the study area (~25–30 km away). For Isle Royale, we used hourly wind speed and temperature data recorded at the weather station on the island (~45 km away). We obtained daily snow depth data for Isle Royale by averaging the values from the two nearest weather stations on the mainland (~50 km away). For Yukon, we used hourly wind speed and temperature, and daily snow depth recorded at the nearest weather station to the study area (~40 km away).

For the analysis of hare and predator space use, we obtained data on the following landscape variables for each site: forest cover type, percent canopy cover, tree density, and elevation (see Appendix S1: Methods S2 and Table S1). Spatial resolution of the data sources was 30 m, except for elevation and tree density data for Yukon, which we rasterized in 30 × 30 m resolution for spatial consistency. We additionally calculated vector ruggedness measurement (VRM), a terrain ruggedness index based on elevation (Sappington et al., 2007; see Appendix S1: Methods S2).

### Diel activity pattern of hares and their predators

We quantified diel activity patterns of hares and predators by combining hare (accelerometer‐based) and predator activity data (camera‐based) for each site (see Appendix S1: Methods S3). We transformed the clock times into solar times relative to the mean time of sunrise and sunset over the study period using the R (R Core Team, 2023) package *activity* (Rowcliffe, 2023), to account for the overwinter change in night length (see Appendix S1: Methods S3). We then fit a von Mises kernel density function to the data using the R package *overlap* (Ridout & Linkie, 2009).

We quantified the level of nocturnality for predators by dividing the area under the activity density curve during the night by the area under the curve over the entire diel cycle (AUC_night_/AUC_24h_), using the R package *DescTools* (Signorell, 2023). To obtain the mean and 95% CI values of AUC_night_/AUC_24h_, we generated 1000 smoothed bootstrap samples of hare and predator activity data using the R package *overlap* and then fit a von Mises kernel density function to each of the bootstrap samples. Finally, using the AUC values estimated from the bootstrap samples, we calculated the degree of activity overlap with 95% CIs between hares and predators for night and daytime as the area under both hare and predator activity density curves.

To compare hare nocturnality among the sites while accounting for environmental factors, we calculated daily nocturnality (0–1) for each hare and each day by dividing night activity time by daily (over 24 h) activity time. We then analyzed daily nocturnality as a function of site, daytime–nighttime temperature difference (=daytime temperature − nighttime temperature), moonlight (fraction of moonlight illuminated), and Julian date (proxy of night length) using generalized linear mixed‐effects models with a beta distribution using the R package *glmmTMB* (Brooks et al., 2017). We considered daytime–nighttime temperature difference because allocating activity to daytime can be energetically beneficial when temperature is warmer during the daytime relative to the night (Lourens & Nel, 1990). We accounted for the effect of moonlight based on the previous finding that hares decrease the level of nighttime activity under bright moonlight (Studd et al., 2019). We pooled the hare activity data of all sites and considered three 2‐way interaction terms, “temperature difference × site,” “moonlight × site,” and “Julian date × site” as fixed effects to account for potential interaction effects between site and environmental factors. We compared 8 candidate models (1 with no interaction term, 3 with one interaction term, 3 with two interaction terms, and 1 with three interaction terms) using the corrected Akaike information criterion (AIC_c_) for small sample size (Burnham & Anderson, 2002) with the *MuMIn* package in R (Bartoń, 2023). Hare ID was included as a random intercept in all the candidate models.

### Space use of hares and their predators

We used integrated step selection analysis (Avgar et al., 2016) to examine how space use and movement of hares were mediated by predation risk while accounting for variable environmental conditions (see Appendix S1: Methods S4). We created steps from the GPS points using the R package *amt* (Signer et al., 2019) and then resampled the steps at a 4‐h sampling rate with a 2‐h tolerance to account for inconsistent GPS fix rates among the sites. We then created 10 random steps for each observed step with a gamma distribution for step length and a von Mises distribution for turn angles. For the end point of each observed and random step, we (1) assigned time of day, moonlight (only for night steps), temperature, wind speed, and snow depth, and (2) extracted landscape variables (cover type, canopy cover, tree density, elevation, and VRM) within a 50‐m buffer.

We fit a conditional logistic regression model to the step data with step ID as the strata by using the *survival* package in R (Therneau, 2023). We considered a model that included all landscape variables to estimate selection as additive terms as the base model. We then constructed candidate models by adding either one or two 2‐way interaction terms between the landscape variables and between landscape and environmental variables to the base model (see Appendix S1: Methods S4). We then conducted a model comparison for each of our study sites using AIC_c_. All candidate models had the same movement component structure, including the natural logarithm of step length (logSL) and interactions of logSL with previously identified environmental drivers of hare activity (temperature, wind, snow, and moonlight; Shiratsuru & Pauli, 2024). We also included cosine of turn angle (cosTA) and the interaction of cosTA with snow depth in the movement component. All the numeric covariates were standardized by mean‐centering and dividing by their SDs prior to model fitting.

Because hares were strongly nocturnal, we conducted step selection analyses for night and daytime steps separately. We considered each unique combination of hare ID and winter as the sampling unit. We retained the hare ID × winter combinations (1) with ≥25 observed steps for the analysis of night steps and (2) with ≥15 observed steps for the analysis of daytime steps, to maximize sample size. To deal with the limited sample size for Isle Royale and interindividual variation in sample size, we created 1000 bootstrapped samples by randomly sampling 25 (for night step analysis) or 15 observed steps (for daytime‐step analysis) with replacement from each hare × winter combination and then creating 10 random steps for each observed step for each site. We then fit the candidate models to the bootstrapped samples. We identified the top model for night and daytime steps separately for each site as the one with the largest mean AIC_c_ weight, and then calculated the mean and 95% bootstrap CIs for each coefficient from the top model.

To identify spatial drivers of predator activity while accounting for environmental conditions, we fit single‐species occupancy models (MacKenzie et al., 2003) to the camera‐based predator detection data summarized in a 2‐week detection format (see Appendix S1: Methods S4 and Table S2) using the R package *unmarked* (Fiske & Chandler, 2011; Kellner et al., 2023). For Wisconsin, we pooled coyote and red fox detections as canid predators and those of fisher and ermine as mustelid predators. We considered consecutive 2‐week periods as repeated “surveys” at each site and the entire winter as a “season.” We interpreted detection of predator species at a camera site such that the species used landscape features of the site during the sampling period instead of assuming site closure (Burton et al., 2012). We treated ψ as the probability of species occurrence, while interpreting detection probability (*p*) as the intensity of species activity at a site, which can be driven by species density and/or habitat selection (Nickel et al., 2020). Therefore, our inference was focused on interpreting parameter estimates for *p*, assuming that spatial and environmental drivers of *p* are associated with predictable predation risk for prey.

Because we intended to capture the overall patterns of predator space use for each site instead of colonization–extinction dynamics, we used single‐season single‐species occupancy models by treating each unique combination of camera site and winter as a distinct site (see Appendix S1: Methods S4). For each 2‐week survey period, we calculated the average daily temperature and snow depth. For each site, we extracted landscape variables (cover type, canopy cover, tree density, elevation, VRM, and distance to linear features) within a 100‐m buffer. Because our focus was on testing whether the patterns of predator spatial activity could explain hare spatial activity, we adopted the covariate structure from the top hare step selection models for the detection component of the predator occupancy model for each site. We then added predator‐only detection covariates (year and distance to linear features) as fixed effects and site as a random intercept to the detection component of the models. To identify the optimum structure of the occupancy component, we compared three models based on AIC_c_: (1) constant‐occupancy model, (2) model with year as the only occupancy covariate, and (3) model with year and additive effects of all the landscape variables as occupancy covariates. We assessed model fit of the selected occupancy models using the MacKenzie–Bailey test (MacKenzie & Bailey, 2004) in the R package *AICcmodavg* (Mazerolle, 2023). In cases of overdispersion, we corrected SEs of parameter estimates for inference (see Appendix S1: Methods S4). All numeric covariates were standardized by mean‐centering and dividing by their SDs prior to model fitting.

### Movement of hares

To examine if the fundamental pattern of hare movement is different among our study sites, we fit the top night step selection model to the entire observed night step data (observed night steps from all the individuals with ≥25 observed night steps) for each site. We then used the estimated shape parameters to obtain distributions of selection‐free step length and turn angle accounting for the effects of the environmental factors (Avgar et al., 2016).

## RESULTS

We obtained accelerometer data from 29 hares (11 females, 17 males, and 1 unknown sex) in Wisconsin, 15 hares (6 females, 7 males, and 2 unknown sex) in Isle Royale, and 68 hares (47 females and 21 males) in Yukon. These accelerometer data produced 797 hare‐days of activity data for Wisconsin, 532 hare‐days of activity data for Isle Royale, and 2738 hare‐days of activity data for Yukon. We collected GPS location data from 20 hares (8 females, 11 males, and 1 unknown sex) in Wisconsin, 15 hares (6 females, 7 males, and 2 unknown sex) in Isle Royale, and 51 hares (42 females and 9 males) in Yukon, which produced 2760 steps (1551 night and 1209 daytime steps) for Wisconsin, 1302 steps (681 night and 351 daytime steps) for Isle Royale, and 9670 steps (5991 night and 3679 daytime steps) for Yukon. In total, we obtained 94 predator detections (43 canid detections and 51 mustelid detections) over 712 camera‐weeks in Wisconsin, 394 predator (fox) detections over 1295 camera‐weeks in Isle Royale, and 442 predator detections (346 lynx detections and 96 coyote detections) over 2276 camera‐weeks in Yukon (Appendix S1: Table S2).

### Diel activity pattern of hares and their predators

**FIGURE 1:**
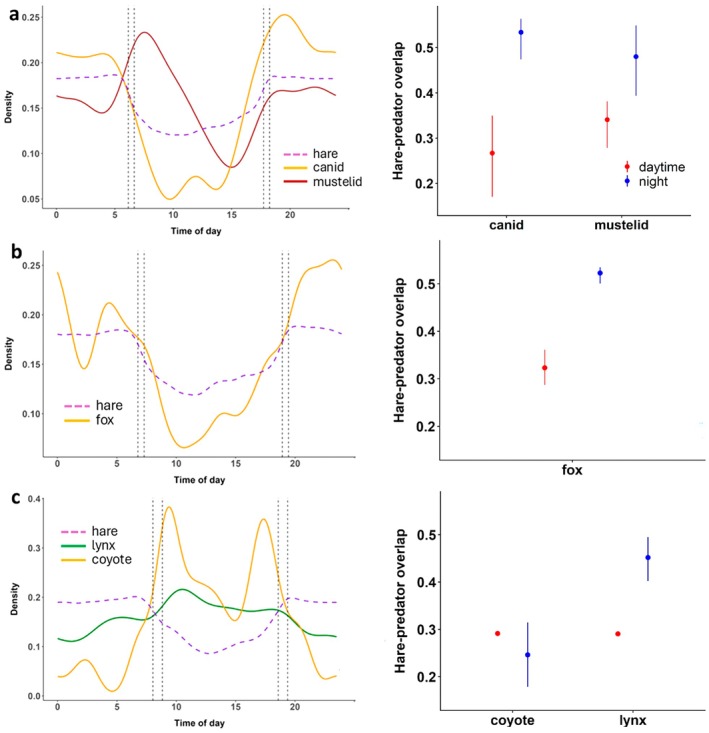
Probability density (von Mises kernel density estimation) of snowshoe hare *Lepus americanus* and predator activity over the diel cycle and resulting hare–predator activity overlap in (a) Wisconsin, (b) Isle Royale, and (c) Yukon. Predator species were canids (coyote *Canis latrans* and red fox *Vulpes vulpes*) and mustelids (fisher *Pekania pennanti* and ermine *Mustela erminea*) in (a), red fox in (b), and lynx *Lynx canadensis* and coyote in (c). Vertical dotted lines represent the average time of sunrise, sunset, and civil dawn/dusk in each site over the study period. Error bars represent 95% bootstrap CIs, and note that the CIs for daytime hare–predator activity overlap are very small in (c).

**FIGURE 2:**
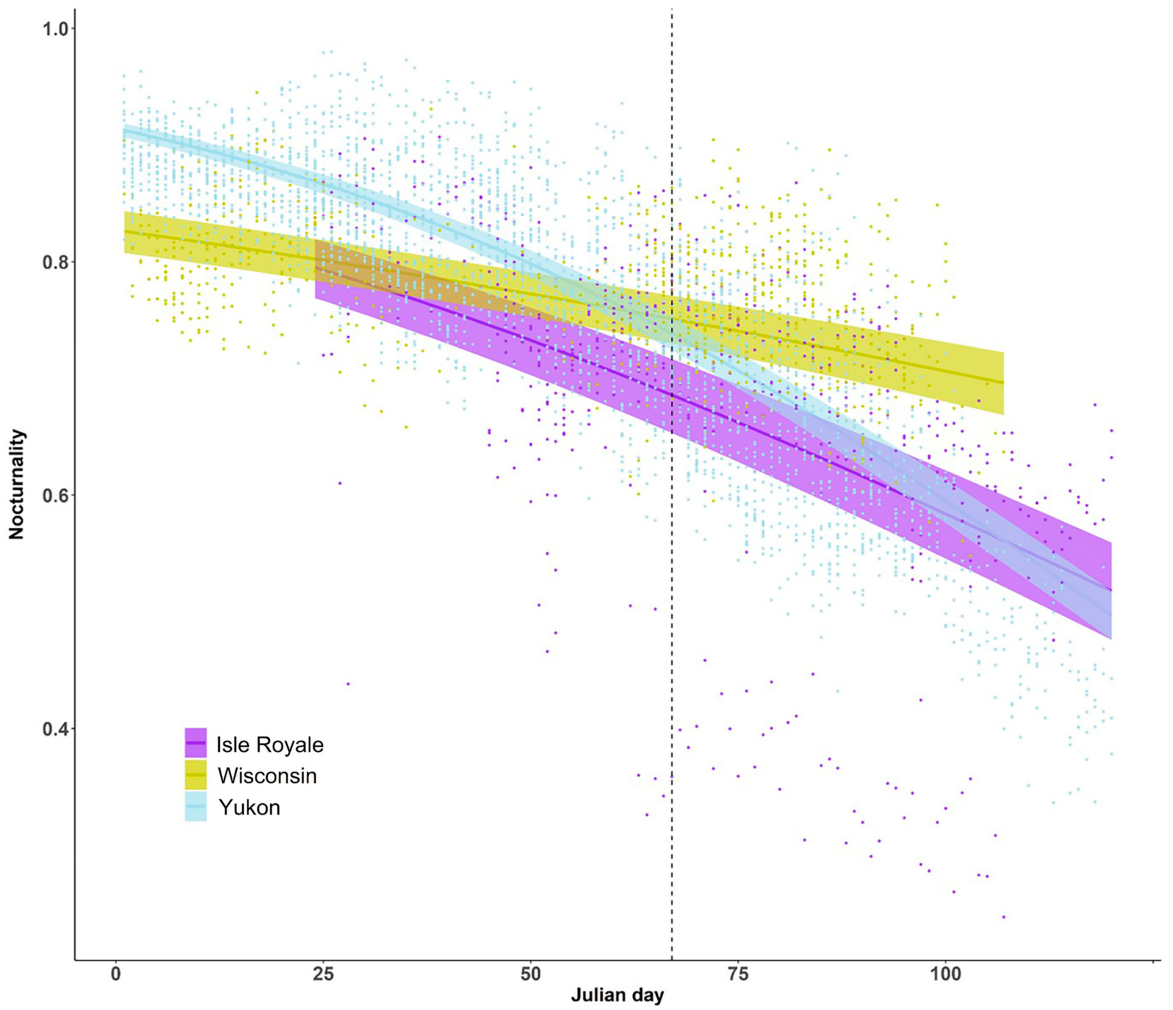
Changes in nocturnality of snowshoe hare *Lepus americanus* (proportion of night activity time over daily activity time for each hare for each day) with Julian day predicted by the generalized linear mixed‐effects model accounting for temperature and moonlight (day–night temperature difference = 0 and moonlight = 0.5). Predicted values are presented with 95% CIs, and the vertical dashed line represents Julian day = 68 (March 8) on which night length is equal across the sites. Small dots represent raw data points.

Hares were largely nocturnal, exhibiting similar diel activity patterns across the three sites (Figure 1). Nocturnality of hares was predicted to be slightly higher in Wisconsin (0.75, 95% CI = [0.73, 0.77]) and Yukon (0.73, [0.72, 0.75]) compared to Isle Royale (0.68, [0.65, 0.71]), after controlling for daytime–nighttime temperature difference, moonlight, and night length (Figure 2). Warmer temperatures during the daytime compared to the night resulted in decreased nocturnality of hares only in Yukon (Appendix S1: Figure S2, Table S3). Hares reduced their nocturnality with shortening night length (Julian date) at all sites, and this reduction in nocturnality with Julian date was larger in Isle Royale and Yukon (Figure 2; Appendix S1: Table S3). Moonlight also negatively affected hare nocturnality, but this effect was weak and did not vary among the sites (Appendix S1: Figure S2, Table S3).

In Wisconsin, canids were more active at night (AUC_night_/AUC_24h_ = 0.650, [0.534, 0.772]), whereas mustelids were equally active during the daytime and night (AUC_night_/AUC_24h_ = 0.505, [0.401, 0.611]) (Figure 1a). Hare activity overlap with their predators was higher during the night, but hares did not appear to prioritize avoiding one over the other (Figure 1a). In Isle Royale, foxes were nocturnal (AUC_night_/AUC_24h_ = 0.609, [0.568, 0.650]), resulting in a higher level of hare–fox activity overlap at night (Figure 1b). In Yukon, coyotes were diurnal (AUC_night_/AUC_24h_ = 0.253, [0.183, 0.325]), whereas lynx were active over the entire diel cycle (AUC_night_/AUC_24h_ = 0.452, [0.404, 0.497]) (Figure 1c). Hare–lynx and hare–coyote activity overlap was comparable during the daytime. However, hare–lynx activity overlap was substantially higher than hare–coyote activity overlap at night (Figure 1c).

### Space use of hares and their predators

**FIGURE 3:**
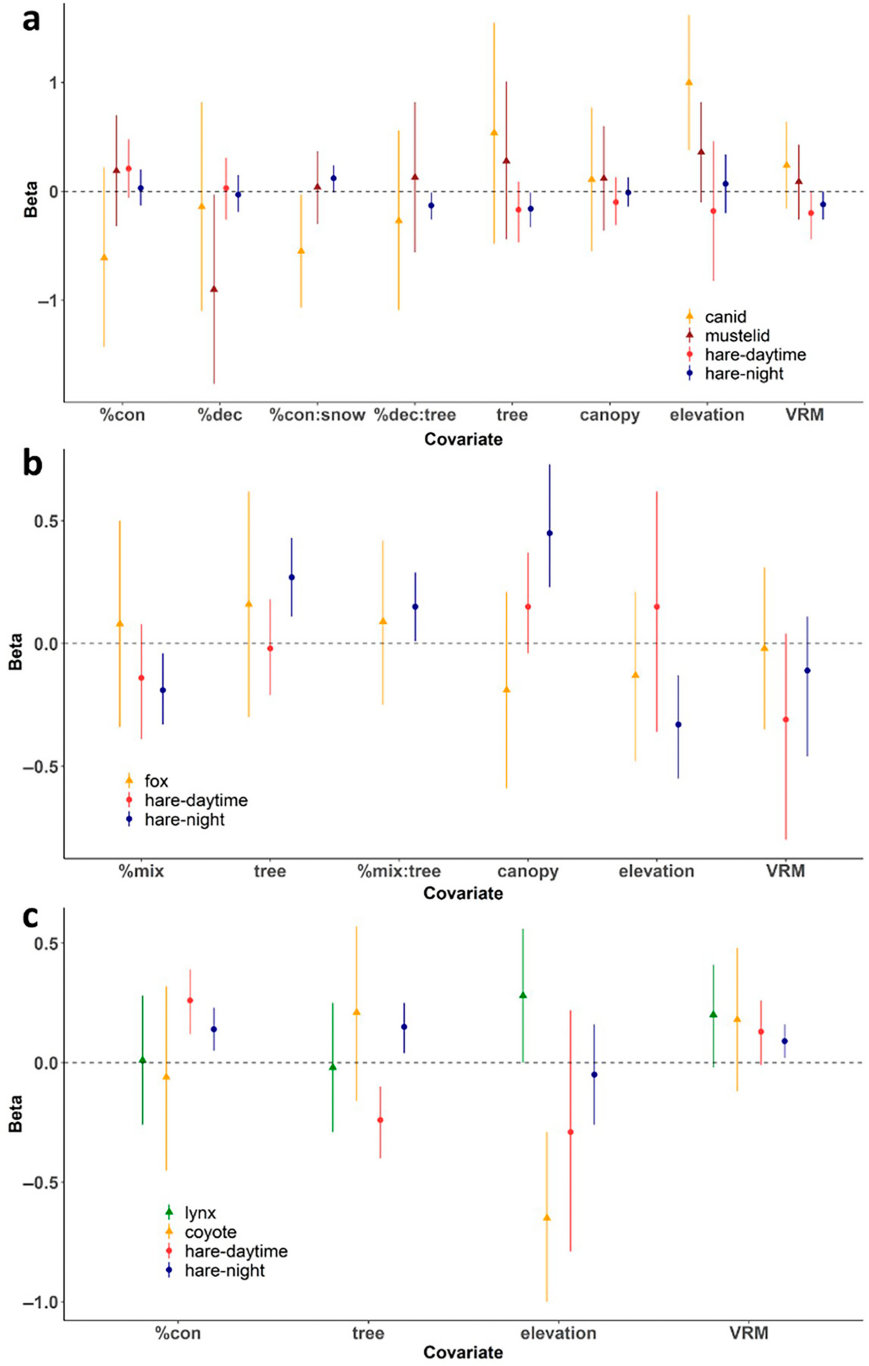
Effects of landscape covariates and their interactions with environmental covariates on snowshoe hare *Lepus americanus* and predator space use in (a) Wisconsin, (b) Isle Royale, and (c) Yukon. Predator species were canids (coyote *Canis latrans* and red fox *Vulpes vulpes*) and mustelids (fisher *Pekania pennanti* and ermine *Mustela erminea*) in (a), red fox in (b), and lynx *Lynx canadensis* and coyote in (c). Beta on *y*‐axis represents coefficient estimates demonstrating the effects of covariates on site selection of hares (selection coefficients estimated by integrated step selection analysis [iSSA] models) and predators (detection coefficients estimated by occupancy models), with positive and negative values indicating selection and avoidance, respectively. All the landscape and environmental covariates were standardized in hare iSSA and predator occupancy models, and error bars represent 95% CIs. %con is percent coniferous forests, %dec is percent deciduous forests, %mix is percent mixed forests, canopy is percent canopy cover, tree is tree density, VRM is vector ruggedness measurement, and snow is daily snow depth.

In Wisconsin, hares avoided high tree density and deciduous forests with high tree density at night (Figure 3a; Appendix S1: Table S4). Hares marginally increased selection for coniferous forests at night when snow was deeper (Figure 3a; Appendix S1: Table S4). Additionally, hares marginally avoided more rugged terrain both during the daytime and night (Figure 3a; Appendix S1: Table S5). Canids decreased the selection for coniferous forests with deeper snow, which was opposite to the response of hares at night (Figure 3a; Appendix S1: Table S6). Mustelids avoided deciduous forests regardless of tree density (Figure 3a; Appendix S1: Table S6).

On Isle Royale, hares avoided mixed forests and higher elevation and selected for higher tree density and denser canopy cover at night (Figure 3b; Appendix S1: Table S7). During the daytime, hares did not select for/against any habitat features (Figure 3b; Appendix S1: Table S8). None of the habitat features affected space use of foxes (Figure 3b; Appendix S1: Table S9).

In Yukon, hares selected for coniferous forests and more rugged terrain both during the day and night (Figure 3c; Appendix S1: Tables S10 and S11). Hares also selected for higher tree density at night, but they showed the opposite response during the daytime (Figure 3c; Appendix S1: Tables S10 and S11). Lynx marginally selected for higher elevation, whereas coyotes selected for lower elevation (Figure 3c; Appendix S1: Table S12).

### Movement of hares

**FIGURE 4:**
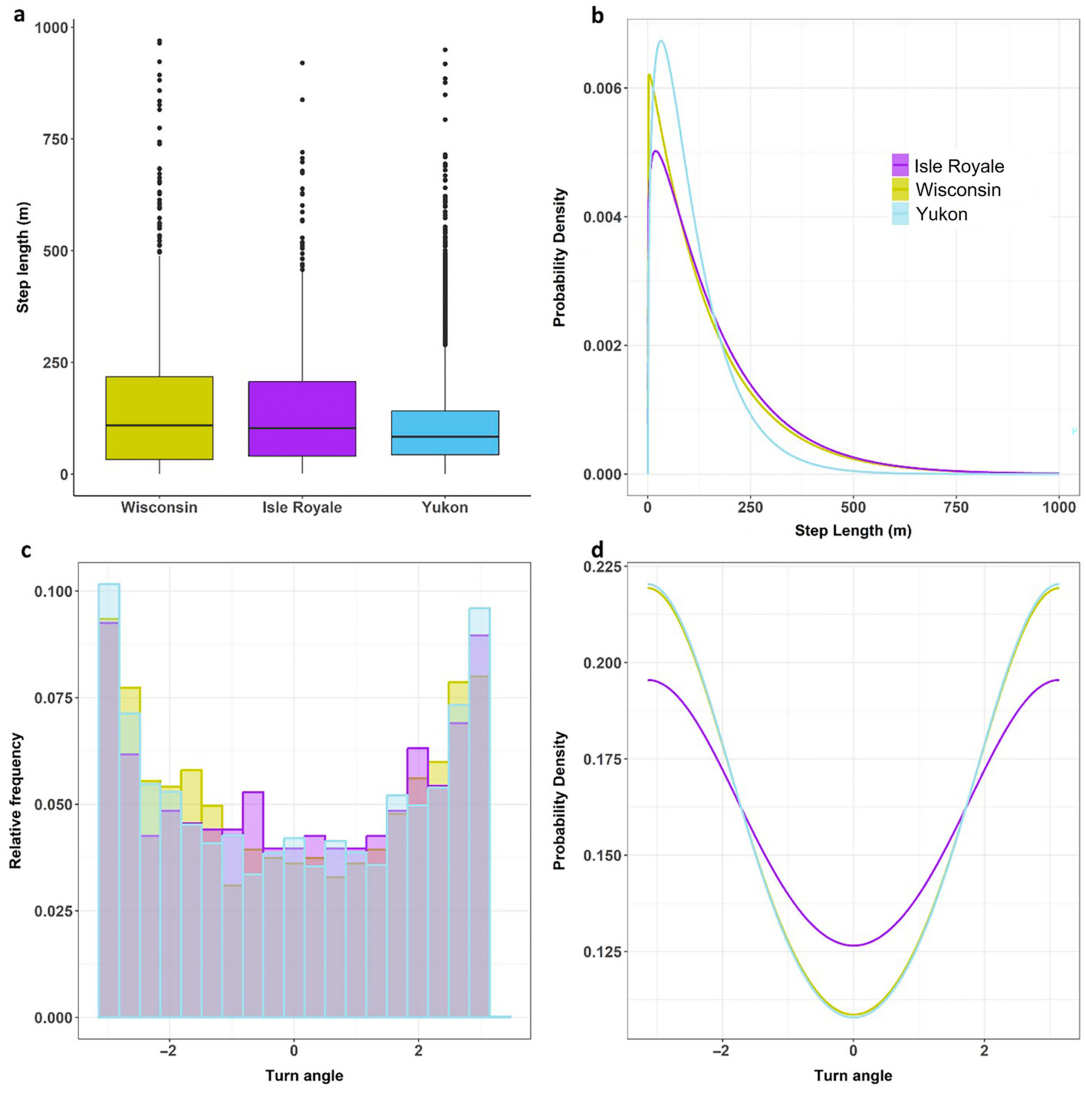
(a) Observed night step length, (b) distributions of selection‐free night step length (on the average environmental conditions) obtained from the top integrated step selection analysis (iSSA) model, (c) relative frequency of observed night turn angle, and (d) distributions of selection‐free night turn angle (on the average environmental conditions) obtained from the top iSSA model, of snowshoe hares *Lepus americanus* in Wisconsin, Isle Royale, and Yukon. Step length represents distance (in meters) hares traveled during 4‐h periods (with a 2‐h tolerance).

Hares exhibited shorter night steps in Yukon (median = 83.8 m, SE = 1.25) compared to Wisconsin (median = 109.3 m, SE = 3.70) and Isle Royale (median = 102.8 m, SE = 5.56) (Figure 4a). This pattern remained the same when the effects of the environmental factors were accounted for by the step selection models (Figure 4b). Also, hares in Wisconsin and Yukon exhibited larger turn angles compared to Isle Royale (Figure 4c), with this pattern being held when the environmental conditions were controlled for (Figure 4d). Snow depth decreased hare movement rate at all sites and increased hare turn angle (decreased cosTA) in Yukon (Appendix S1: Figure S3).

## DISCUSSION

Our hypothesis—that the spatiotemporal patterns of hare behaviors would diverge across sites due to the difference in the species composition and traits of predators—was only partially supported. Hares were only slightly less nocturnal on Isle Royale, where the single generalist predator (fox) was nocturnal, compared to the other sites. Moreover, hares exhibited similar diel activity patterns across all sites, and thus, hare–predator temporal overlap was primarily determined by predator diel activity patterns. Patterns of spatial risk avoidance of hares were moderately different across the sites, which may be partially attributable to the differences in predator species composition and traits, though there were confounding factors we could not account for. Hares in multi‐predator systems either exhibited a pattern of space use opposite to that of more lethal predators (in Wisconsin with multiple generalists) or switched the pattern of space use between daytime and nighttime (in Yukon with a single specialist and a single generalist). Additionally, hares exhibited shorter and less directional movements where the ambush predator was present (in Yukon with lynx). Our findings demonstrate that prey behaviors related to spatiotemporal risk avoidance may exhibit slight geographic variation, but certain behavioral axes such as diel activity patterns may be more fixed and thus conserved across ecosystems compared to others such as spatial behaviors.

Ecological studies have been increasingly examining diel activity patterns of predators and prey as an indicator of their behavioral interactions (Monterroso et al., 2013; Murphy et al., 2021). Some have reported alteration of diel activity patterns by prey in response to changes in predation risk, such as increases/declines of predator populations (e.g., Cunningham et al., 2019). However, we observed that the nocturnality of hares was only slightly lower (<10%) on Isle Royale, where daytime risk was the lowest; the amount of daily activity (10–12 h in winter; Peers et al., 2020) allocated to the nighttime would only be <1 h less for Isle Royale hares compared to the other sites. Additionally, overall diel activity patterns of hares were highly comparable among our study sites. Our finding is consistent with previous findings that activity responses of prey to light levels can be inflexible and conserved due to evolutionary constraints on the underlying endogenous rhythms (Kronfeld‐Schor et al., 2013; Kronfeld‐Schor & Dayan, 2003; Prugh & Golden, 2014). This is corroborated by the fact that the negative effect of moonlight on the nocturnality of hares was also weak and consistent across the sites. Moreover, hare nocturnality decreased with shortening night length (Julian date) at all sites, and the rate of this reduction in hare nocturnality with Julian date increased with latitude. This illustrates that diel activity patterns of hares are largely determined by the amount of time available for nocturnal activities (Gilbert et al., 2023). Therefore, prey may not be capable of fundamentally altering their diel activity patterns (Kronfeld‐Schor & Dayan, 2003), and thus, switching the pattern of habitat use among the diel phases may be the optimal way for them to minimize risk (Kohl et al., 2019; Smith et al., 2019). In rapidly changing environments, animals are altering the spatiotemporal patterns of their behaviors to optimize fitness (Gilbert et al., 2023; Guiden et al., 2019), which can ultimately alter predator–prey interactions. Our study provides insights into this active area of research by addressing potentially limited capacity of prey in altering their diel activity patterns, though we also note that this pattern may be mediated by prey traits (Burton et al., 2024; Wirsing et al., 2021).

Hares in Wisconsin marginally increased their selection for coniferous forests with deeper snow at night, which was the opposite to the response by canid predators. Canids are considered as more lethal predators than mustelids for hares in Wisconsin (Keith et al., 1993; Sievert & Keith, 1985). Therefore, this finding matches our expectation that hares would prioritize avoiding more lethal predators in multi‐predator systems (Lima, 1992; Thaker et al., 2011) at times of day when multiple predators are active. In Yukon, a direct negative association between hare and lynx/coyote selection was not observed for any landscape features, regardless of time of day. However, hares selected for higher tree density at night, whereas they avoided higher tree density during the daytime. It is noteworthy that previous research demonstrated that lynx chase hares in more open spruce forests while coyotes prefer denser spruce forests in hunting hares (Murray et al., 1995). Moreover, the ratio of coyote predation to lynx predation is much higher during the daytime than at night (Shiratsuru et al., 2023). Therefore, hares may be prioritizing avoiding lynx predation at night and doing the opposite during the daytime. This is consistent with the finding that prey can optimize their spatiotemporal risk avoidance by altering their space use depending on the time of day in multi‐predator systems where different predators are active at different times of day (Kohl et al., 2019). Hares in Isle Royale exhibited selection for and against many of the landscape features at night when foxes are active, but none of these landscape features impacted fox activity. This implies that spatial risk avoidance may not be the primary driver of space use of prey, even in single‐predator systems, when the predator shows no clear patterns of habitat selection and thus is spatially unpredictable. However, it is possible that the differences in the temporal resolution of the space use data between hares (step selection analysis with 4‐h sampling intervals) and predators (occupancy modeling with 2‐week detection summary) may have limited our ability to fully unveil the patterns of their space race. Additionally, resource availability is an important driver of prey space (e.g., Majchrzak et al., 2022) besides predation risk. Therefore, we cannot rule out the possibility that the divergent patterns of space use of hares across the sites may be partly driven by the inter‐site differences in forest/vegetation types.

Predator hunting modes can mediate the effects of rate and directionality of prey movement on predator–prey encounter rates (Scharf et al., 2006). It is generally acknowledged that movement rates of predators and prey are the primary determinants of their encounter rates, and movement rates of predators tend to be higher than those of prey and thus be more important in many cases (Hutchinson & Waser, 2007). However, the relative importance of rate and directionality of prey movement theoretically increases when they interact with ambush predators compared to when interacting with active predators (Avgar et al., 2008; Dell et al., 2014; Pawar et al., 2012). Therefore, we expected that hares in Yukon, where there is an ambush specialist (lynx), would generally show lower movement rates and a larger turn angle (higher tortuosity). Indeed, step length was shorter and turn angle was larger in Yukon compared to the other sites. Additionally, hares in Yukon also increased turn angle with deeper snow. Considering that lynx in Yukon tend to decrease the level of activity when snow is deep (Menzies et al., 2022), they may be more reliant on ambushing for catching prey when snow is deep, making it beneficial for hares to decrease directionality of their movement. Interestingly, turn angle distribution of Wisconsin hares was similar to that of Yukon hares. This is possibly because there are bobcats in Wisconsin, which can be considered as ambush predators (Litvaitis et al., 1986), though they were very rare in our study site (only 1 detection over 712 camera‐weeks in winters 2022–2023). It is noteworthy, however, that resource patchiness and patch density can also affect the movement patterns of herbivorous prey (de Knegt et al., 2007; Zollner & Lima, 1999), which may partially explain the observed geographic variation in hare movement.

Across their distributional range, snowshoe hares exhibit variation in their morphology and population ecology. For example, hare populations from Wisconsin to Alaska exhibit a positive linear relationship between body mass and latitude (body mass increases by 130 g with an increase of 10° in latitude), which is attributed to the energetic benefit of larger body size at higher latitude (Gigliotti et al., 2020). Therefore, smaller hares may need to shift their balance of the energy‐safety trade‐off towards starvation avoidance compared to larger hares in winter (Preisser & Orrock, 2012). If that were the case, we would have expected less antipredator responses among hares at lower latitudes. However, we did not detect a clear attenuation of antipredator behaviors of hares from Yukon to Isle Royale and Wisconsin. Additionally, population dynamics of hares vary across their range. In the core of their distribution, including Yukon, hares exhibit strong cyclical dynamics with a 10‐year periodicity (Krebs et al., 2001). In contrast, along their southern range boundary in Wisconsin, snowshoe hares are weakly cyclic or have become noncyclic (Chandross & Pauli, 2025). High predation pressure from diverse generalist predators, habitat fragmentation, and declining snow conditions have all been implicated as mechanisms underlying the relatively stable, low population size of hares in the south (Buehler & Keith, 1982; Keith et al., 1993). In ecosystems where hare and predator population densities fluctuate annually (Yukon), the spatiotemporal pattern of risk likely varies with changes in relative abundances and behavioral patterns of predators (Hodges et al., 2001; Murray et al., 1994). This could result in annual variation in the patterns of spatiotemporal risk avoidance by hares (Majchrzak et al., 2022), though we could not test that because we pooled multi‐winter data on hare and predator behaviors for each site to compare the overall and population‐level trends of hare behaviors among the study sites. Thus, several differences exist across our study sites, including, but not limited to, the variation in predator traits and composition, all of which can drive geographic patterns of antipredator behaviors of hares. Nevertheless, our study suggests limited geographic variation in multiple antipredator behaviors of hares, despite the cross‐site differences.

Although we could not test with our data, there are alternative mechanisms that may be driving the behavioral patterns we observed in hares. For example, lack of data prevented us from assessing how predation mortality rate and resource availability were different among our study sites. Future studies conducting a cross‐site comparison of prey behaviors with access to such data could better disentangle the effects of food availability from predation pressure (McNamara & Houston, 1987). We also note that the timing (in years) of data collection was different among the sites, which might have amplified the confounding effect of annual variation in spatiotemporal risk on the observed patterns of hare behaviors. Individual variation in prey behaviors related to risk avoidance (Mazza et al., 2019) and habitat selection (Viana et al., 2018) has also been reported by previous studies. Therefore, a more comprehensive understanding of the role of risk in driving prey behaviors requires consideration of annual and individual variation.

The importance of predation risk in affecting prey behaviors and thereby driving species interactions and overall community dynamics is well acknowledged (Creel, 2018; Lima, 1998). How the patterns of these risk effects are mediated by traits of predators and prey as well as environmental conditions is now attracting more attention from community ecologists (Wirsing et al., 2021). By comparing multiple behavioral axes of the same prey species across different ecosystems while accounting for environmental conditions, we examined geographic variation in prey behaviors in relation to the species composition and traits of predators. Although we could not account for all the potential confounding factors, our study demonstrated that flexibility and resultant geographic variation in antipredator behaviors of hares differed between diel activity patterns and spatial behaviors. This result addresses that the degree of evolutionary persistence of prey behaviors related to risk avoidance varies among different behavioral axes (Blumstein, 2006). Notably, a behavioral trait of prey is more likely to persist when the fitness benefit exceeds the maintenance cost (Blumstein, 2006). Therefore, reactive behavioral responses of prey triggered upon immediate predator encounters (Creel, 2018) may be more inflexible and conserved across ecosystems compared to proactive responses, which need to be tested by future studies. Finally, we also recommend future studies to explicitly examine how predator traits (e.g., morphological adaptations and sensory modalities) interact with environmental conditions to drive geographic variation in antipredator behaviors. These cross‐site intraspecific comparisons of multiple behavioral axes of prey will provide valuable insights into the mechanism underlying spatiotemporal patterns of prey behaviors and resulting predator–prey interactions.

## AUTHOR CONTRIBUTIONS

Shotaro Shiratsuru, Stan Boutin, and Jonathan N. Pauli conceived and designed the study. Shotaro Shiratsuru, Yasmine N. Majchrzak, Michael J. L. Peers, Emily K. Studd, Alice J. Kenney, and Hailey M. Boone led data collection. Logistical support was provided by Jonathan N. Pauli, Stan Boutin, Dennis L. Murray, Jerrold L. Belant, and Mark Romanski. Shotaro Shiratsuru performed the analyses. Shotaro Shiratsuru and Jonathan N. Pauli drafted the manuscript with input from all authors.

## CONFLICT OF INTEREST STATEMENT

The authors declare no conflicts of interest.

## Supporting information


Appendix S1.

## Data Availability

Data and code (Shiratsuru et al., 2026) are available in Figshare at https://doi.org/10.6084/m9.figshare.29917115.v1.
