# Supplementary material for: A cross‐site comparison reveals limited geographic variation in risk avoidance of snowshoe hares
Source: Ecology. 2026 Apr 10;107(4):e70378. doi: 10.1002/ecy.70378 (PMC13067815; doi:10.1002/ecy.70378)
Supplement: Supplementary file 1 — Appendix S1. [file ECY-107-e70378-s001.pdf]

Appendix S1

A cross-site comparison reveals limited geographic variation in risk avoidance of snowshoe hares

Shotaro Shiratsuru, Emily K. Studd, Michael J. L. Peers, Yasmine N. Majchrzak, Alice J. Kenney, Dennis L. Murray, Mark Romanski, Jerrold L. Belant, Hailey M. Boone, Stan Boutin, Jonathan N. Pauli

Ecology

Methods S1-Study area

In Wisconsin, primary habitat types are conifer-deciduous mixed forests (e.g., *Picea glauca*, *Larix laricina*, *Populus tremuloides*, *Populus balsamifera*) with understory shrubs (*Salix* spp. and *Alnus* spp.) (Ribic et al. 2017). In Isle Royale, dominant habitat types are northern hardwood forests (e.g., *Betula* spp., *Acer* spp., and *Quercus* spp.), conifer swamps (*Thuja occidentalis*) and conifer-deciduous mixed forests (e.g., *Picea* spp. and *Abies* spp.) (Sanders and Kirschbaum 2023, Rodriguez Curras et al. 2024). In Yukon, primary habitat types are conifer-dominant forests (*Picea glauca*) with understory shrubs (*Salix glauca* and *Betula glandulosa*) (Boonstra et al. 2018).

Predators in Wisconsin are primarily mammalian generalists (coyote, red fox, fisher and ermine, bobcat), though avian predators (e.g., great horned owls *Bubo virginianus* and potentially northern goshawk *Accipiter gentilis*) are also present (Sievert and Keith 1985, Wilson et al. 2022). On Isle Royale, red fox, a generalist, is the only primary predator for hares (Johnson 1970) and predation by avian predators (Shelton 1975) or by American martens *Martes americana* (Lacin Alas et al. 2025) are not very common. Primary predators of hares in Yukon are lynx, a specialist, and coyote, a generalist, while avian predators including northern goshawks and great horned owls are secondary (Boutin et al. 1986).

Methods S2-Data collection

Cameras (48 cameras in 2022 and 29 cameras in 2023; Bushnell Outdoor Products, Cody Overland Park, Kansas, USA) were deployed at randomly selected locations within 100–300 m from the forest road throughout the study area (~400 km^2 area) with 1,042 m spacing on average (473-4,090 m) in Wisconsin (Figure S1). We utilized predator detection data from the cameras (40 cameras in 2021 and 37 cameras in 2022; Stealth Cam DS4K, Irving, Texas, USA) deployed in the western part of the island (~180 km^2 area) that were 1,192 m apart on average (332-2,671 m) for Isle Royale (Figure S1, see Boone et al. 2025 for details). These cameras were deployed within 50 m of the trails, but the island was not open to the public during the winter months. For Yukon, we used predator detection data obtained by the cameras (74 cameras in 2018 and 68 cameras in 2019; Reconyx Inc., Holmen, Wisconsin, USA) deployed at locations with 857 m spacing on average (242-1,901 m) throughout the study area (~200 km^2 area) that were randomly selected with respect to the primary vegetation types in the area (Figure S1, see Kenney et al. 2024 for details). These cameras were on average 643 m (25-3,190 m) from the nearest linear feature (either the highway or the local road intensively used). For all the study sites, photo series of the same predator species at the same camera site with a time lag of ≥30 min were considered independent.

Hares were live-captured using Tomahawk live-traps (Tomahawk Live Trap Co., Tomahawk, Wisconsin, USA), and individual ID on the ear-tag and sex were recorded upon each

capture in all sites. Hares were opportunistically trapped in Wisconsin (~40 km^2 area, Figure S1) and Isle Royale (~6 km^2 area, Figure S1), while hares were captured on five 600 m × 600 m trapping grids in Yukon (~10 km2 area, Figure S1). Collar weight was <5% of hare body mass. Accelerometer data were classified into not moving, foraging (feeding and travel with one hop), hopping and sprinting with a hierarchal decision tree for hares developed by Studd et al. (2019), which was shown to have high accuracy (97.0 %) in classifying an active state of hares (Studd et al. 2019).

We obtained meteorological data including wind speed, air temperature and daily snow depth for each study site. For Wisconsin, we used wind speed and temperature data recorded every 20 min as a part of the Automated Surface Observing Systems (ASOS) at the nearest available weather station to the study area (~ 30 km away; Taylor County Airport, 45.10° N, 90.30° W), and daily snow data recorded at the nearest available weather station (~ 25 km away; Mather 3NW USC00475164, 44.17° N, 90.33° W). For Isle Royale, we used wind speed and temperature data recorded every 60 min at the nearest available weather station to the study area (~ 45 km away; OJIM4, 48.11° N, 88.61° W) which was on the eastern end of the island. We obtained daily snow depth data for Isle Royale by taking the average of the values from the two nearest weather stations on the mainland (~ 50 km away; THUNDER BAY CS, 48.37° N, 89.33° W, and USC00213296, 47.97° N, 89.69° W), because no weather stations on the island recorded snow data. We used hourly wind speed and temperature data and daily snow depth data recorded at the nearest available weather station to the study area (~ 40 km away; HAINES JUNCTION, 60.77° N, 137.58° W) for Yukon. We acknowledge that these data may not precisely capture the microscale meteorological conditions at the exact locations of the study sites, but we believe that relative temporal variations in the meteorological conditions were still well reflected.

For the analyses of hare and predator space use, we obtained data on the following landscape variables for each site; forest cover type (Wisconsin Department of Natural Resources 2016, Dewitz 2021, Hermosilla et al. 2022), percent canopy cover (USDA Forest Service 2023, Matasci et al. 2018), tree density (Riley et al. 2022, Government of Yukon, Geomatics Yukon 2025), and elevation (US Geological Survey 2021, Natural Resources Canada 2013). Spatial resolution of the data sources was 30 m, except for elevation (0.75 arc-second) and tree density data (polygons projected at a scale of 1:5,000) for Yukon, which we rasterized in 30 × 30 m resolution to assure spatial consistency. We calculated Vector Ruggedness Measurement (VRM) as the terrain ruggedness index (Sappington et al. 2007) over a 3 × 3 neighborhood centered on each cell based on the elevation data for each site, using the *vrm* function of the *spatialEco* package (Evans and Murphy 2023) in R (R Core Team, 2023).

Methods S3-Diel activity pattern of hares and predators

We quantified diel activity patterns of hares and predators, by combining hare (accelerometer-based) and predator activity data (camera-based detections) for each site. We summarized

accelerometer-based hare activity data in 5-minute time windows and then retained datapoints with the number of seconds within five minutes that a hare spent in an active state > 0. For predators, we considered that an animal was in an active state when detected by a camera. We transformed the clock times (as radians) into solar times relative to the mean time of sunrise and sunset over the study period (see Vazquez et al. 2019 for details) using the *transtime* function in the R package *activity* (Rowcliffe 2023), to account for the overwinter change in night length. We then fit a von Mises kernel density function to the data using the *densityFit* function of the R package *overlap* (Ridout and Linkie 2009).

Methods S4-Space use of hares and their predators

We used integrated step selection analysis (Avgar et al. 2016) to examine how space use and movement of hares were mediated by predation risk while accounting for variable environmental conditions. We created steps (straight lines between the two consecutive GPS points) from the GPS points using the *make_track* function of the *amt* package in R (Signer et al. 2019) and then resampled the steps at a 4-h sampling rate with 2-h tolerance to deal with the inconsistent GPS fix rates among the sites. To remove potential erroneous steps, we only retained the observed steps with step length ≤ 1000 m. We considered steps that ended at night or dawn/dusk as night steps and the ones ended during the daytime as daytime steps. We then created ten random steps for each observed step using the *random_steps* function of the *amt* package in R with gamma distribution for step length and a von Mises distribution for turn angles. For the end point of each observed and random step, we (1) assigned time of day (day or night), fraction of moonlight illuminated (only for night steps), temperature, wind speed (km/h) and snow depth (cm), and (2) extracted landscape variables within 50-m buffer. We assigned the most temporally proximate wind speed and temperature values to each data point. Landscape variables included percent cover type (two dominant cover types among the hare GPS locations for each site; coniferous and deciduous forests in Wisconsin, woody [predominantly coniferous] wetland and mixed forest in Isle Royale, and coniferous forest and shrub in Yukon), mean percent canopy cover, mean tree density (number of trees per hectare), mean elevation and mean VRM.

We fit a conditional logistic regression model to the step data with step ID as the strata by using the *survival* package in R (Therneau 2023). We considered a model that included all landscape variables to estimate selection as additive terms as the base model. We constructed candidate models by adding either one or two two-way interaction terms between the landscape variables as well as between landscape and environmental variables to the base model. We considered the two-way interactions previously identified as important drivers of hare behaviors (Hik 1995, Studd et al. 2019, Shiratsuru et al. 2024); (1) cover type × tree density, (2) cover type × snow, (3) tree density × snow, (4) cover type × moonlight (only for night steps) and (5) canopy cover × moonlight (only for night steps). We did not include covariates exhibiting potential multicollinearity ($|r| \geq 0.7$) in the same model (Dormann et al., 2013). This resulted in (1)

inclusion of a single percent cover type per model for Isle Royale and Yukon and (2) inclusion either of percent coniferous forest and canopy cover per model for Yukon. Additionally, we did not include canopy cover and tree density in the models with percent shrub as the cover type for Yukon, because these variables are irrelevant in shrub habitat (see Supplementary Tables for the list of the candidate models for each study site). We then conducted a model comparison for each of our study sites using AICc. All the candidate models had the same structure of the movement component, which included the natural logarithm of step length (log*SL*) and interactions of log*SL* with previously identified environmental drivers of hare activity (temperature, wind, snow and moonlight; Shiratsuru et al. 2024). We also included cosine of turn angle (cos*TA*) and the interaction of cos*TA* with snow depth in the movement component. All the numeric covariates were standardized by mean-centering and dividing by their standard deviations prior to model fitting.

To examine spatial and environmental drivers of predator activity, we fit single-species occupancy models (Mackenzie et al. 2003) to the camera-based predator detection data summarized in a two-week detection format using the R package *unmarked* (Fiske and Chandler 2011, Kellner et al. 2023). For Wisconsin, we pooled detections of coyote and red fox as canid predators and those of fisher and ermine as mustelid predators. We assigned NA in the detection history when a camera operated ≤ 10 days in a two-week sampling period. We considered consecutive camera-trapping weeks (i.e., camera weeks) as repeated "surveys" at each camera site and the entire winter as a "season". We interpreted detection of predator species at a camera site such that the species used landscape features of the site during the sampling period instead of assuming site closure (i.e., sites are permanently occupied by the species) (Burton et al. 2012). Accordingly, also considering larger spatial scale of predator movement relative to that of our camera deployment, we treated ψ as the probability of species occurrence while interpreting that detection probability (p) represents intensity of species activity at a site which can be driven by density and/or habitat selection of the species (Nickel et al. 2020). Therefore, our inference was focused on interpreting parameter estimates for p, assuming that spatial and environmental drivers of p are associated with predictable predation risk for prey.

Because we intended to capture the overall patterns (i.e., across multiple winters) of predator space use and activity for each site instead of colonization-extinction dynamics, we used single-season single-species occupancy models by treating each unique combination of camera site and winter as a distinct site (e.g., Fuller et al. 2016). We included year as a categorical fixed effect and camera site as a random intercept in the detection component of the models. For each two-week survey period, we calculated the average value of daily temperature and daily snow depth. For each site, we extracted landscape variables within 100-m buffer (considering larger spatial scale of predator space use relative to hares). Landscape variables included percent cover type (two dominant cover types among the hare GPS locations for each site), mean percent canopy cover, mean tree density, mean elevation, mean VRM and distance (m) to linear features (only for Wisconsin and Yukon). Because our focus was on testing whether hare spatial activity

could be explained by the patterns of predator spatial activity, we adopted the spatial and environmental covariates (besides wind speed and moonlight which become irrelevant at a two-week time window) from the top hare step selection models for the detection component of the predator occupancy model for each site. We then added predator-only detection covariates (year and distance to linear features) as fixed effects and site as a random intercept to the detection component of the models. To identify the optimum structure of the occupancy component, we compared three models based on AICc; (1) constant-occupancy (null) model, (2) model with year as the only occupancy covariate and (3) model with year and additive effects of all the landscape variables as occupancy covariates. We assessed model fit of the selected occupancy models by the MacKenzie-Bailey test (MacKenzie and Bailey 2004) by the *mb.gof.test* function of the R package *AICcmodavg* (Mazerolle 2023). In cases of overdispersion (c-hat > 1), we corrected standard errors of parameter estimates for inference by multiplying the variance-covariance matrix of the estimates by c-hat value using the *summaryOD* function of the R package *AICcmodavg*. All numeric covariates were standardized by mean-centering and dividing by their standard deviations prior to model fitting.

Literature Cited

Avgar, T., Potts, J. R., Lewis, M. A., & Boyce, M. S. (2016). Integrated step selection analysis: bridging the gap between resource selection and animal movement. *Methods in Ecology and Evolution*, *7*(5), 619-630.

Boone, H. M., Romanski, M., Kellner, K., Kays, R., Potvin, L., Roloff, G., & Belant, J. L. (2025). Recreational trail use alters mammal diel and space use during and after COVID-19 restrictions in a US national park. *Global Ecology and Conservation*, *57*, e03363.

Boonstra, R., Boutin, S., Jung, T. S., Krebs, C. J., & Taylor, S. (2018). Impact of rewilding, species introductions and climate change on the structure and function of the Yukon boreal forest ecosystem. *Integrative zoology*, *13*(2), 123-138.

Boutin, S., Krebs, C. J., Sinclair, A. R. E., & Smith, J. N. M. (1986). Proximate causes of losses in a snowshoe hare population. *Canadian Journal of Zoology*, *64*(3), 606-610.

Burton, A. C., Sam, M. K., Balangtaa, C., & Brashares, J. S. (2012). Hierarchical multi-species modeling of carnivore responses to hunting, habitat and prey in a West African protected area. *PloS one*, *7*(5), e38007.

Dewitz, J. (2021). National land cover database (NLCD) 2019 products. *US Geological Survey*, *10*, P9KZCM54.

Dormann, Carsten F., Jane Elith, Sven Bacher, Carsten Buchmann, Gudrun Carl, Gabriel Carré, Jaime R. García Marquéz et al. "Collinearity: a review of methods to deal with it and a simulation study evaluating their performance." *Ecography* 36, no. 1 (2013): 27-46.

Evans, J. S., Murphy, M. A. (2023). spatialEco. R package version 2.0-2, <https://github.com/jeffreyevans/spatialEco>.

Fiske, I., & Chandler, R. (2011). Unmarked: an R package for fitting hierarchical models of wildlife occurrence and abundance. *Journal of statistical software*, *43*, 1-23.

Fuller, A. K., Linden, D. W., & Royle, J. A. (2016). Management decision making for fisher populations informed by occupancy modeling. *The Journal of Wildlife Management*, *80*(5), 794-802.

Government of Yukon, Geomatics Yukon. (2025). Vegetation Inventory - 5k - Land Cover. Natural Resources Canada, Federal Geospatial Platform. https://osdp-psdo.canada.ca/dp/en/search/metadata/NRCAN-FGP-1-af071d8c-26a7-7518-4ff9-8cd9fe321a40.

Hermosilla, T., Wulder, M. A., White, J. C., & Coops, N. C. (2022). Land cover classification in an era of big and open data: Optimizing localized implementation and training data selection to improve mapping outcomes. *Remote Sensing of Environment*, *268*, 112780.

Hik, D. S. (1995). Does risk of predation influence population dynamics? Evidence from cyclic decline of snowshoe hares. *Wildlife Research*, *22*(1), 115-129.

Johnson, W. J. (1970). Food habits of the red fox in Isle Royale National Park, Lake Superior. *American Midland Naturalist*, 568-572.

Kellner, K. F., Smith, A. D., Royle, J. A., Kéry, M., Belant, J. L., & Chandler, R. B. (2023). The unmarked R package: Twelve years of advances in occurrence and abundance modelling in ecology. *Methods in Ecology and Evolution*, *14*(6), 1408-1415.

Kenney, A. J., Boutin, S., Jung, T. S., Murray, D. L., Johnson, N., & Krebs, C. J. (2024). Motion-sensitive cameras track population abundance changes in a boreal mammal community in southwestern Yukon, Canada. *The Journal of Wildlife Management*, *88*(4), e22564.

Lacin Alas, B., Rodriguez Curras, M., Smith, M. M., Potvin, L. R., Romanski, M. C., & Pauli, J. N. (2025). The repatriation of wolves to Isle Royale alters the foraging of meso-carnivores. *Journal of Mammalogy*, *106*(1), 30-38.

MacKenzie, D. I., & Bailey, L. L. (2004). Assessing the fit of site-occupancy models. *Journal of Agricultural, Biological, and Environmental Statistics*, *9*, 300-318.

MacKenzie, D. I., Nichols, J. D., Hines, J. E., Knutson, M. G., & Franklin, A. B. (2003). Estimating site occupancy, colonization, and local extinction when a species is detected imperfectly. *Ecology*, *84*(8), 2200-2207.

Matasci, G., Hermosilla, T., Wulder, M. A., White, J. C., Coops, N. C., Hobart, G. W., ... & Bater, C. W. (2018). Three decades of forest structural dynamics over Canada's forested ecosystems using Landsat time-series and lidar plots. *Remote Sensing of Environment*, *216*, 697-714.

Natural Resources Canada. (2013). Canadian digital elevation model.

Mazerolle, M. J. (2023). AICcmodavg: Model selection and multimodel inference based on (Q)AIC(c). R package version 2.3.3, <https://cran.r-project.org/package=AICcmodavg>.

Nickel, B. A., Suraci, J. P., Allen, M. L., & Wilmers, C. C. (2020). Human presence and human footprint have non-equivalent effects on wildlife spatiotemporal habitat use. *Biological Conservation*, *241*, 108383.

Ribic, C. A., Donner, D. M., Beck, A. J., Rugg, D. J., Reinecke, S., & Eklund, D. (2017). Beaver colony density trends on the Chequamegon-Nicolet National Forest, 1987–2013. *PLoS One*, *12*(1), e0170099.

Ridout, M. S., & Linkie, M. (2009). Estimating overlap of daily activity patterns from camera trap data. *Journal of agricultural, biological, and environmental statistics*, *14*(3), 322-337.

Riley, K. L., Grenfell, I. C., Shaw, J. D., & Finney, M. A. (2022). TreeMap 2016 dataset generates CONUS-wide maps of forest characteristics including live basal area, aboveground carbon, and number of trees per acre. *Journal of Forestry*, *120*(6), 607-632.

Rodriguez Curras, M., Romanski, M. C., & Pauli, J. N. (2024). The pulsed effects of reintroducing wolves on the carnivore community of Isle Royale. *Frontiers in Ecology and the Environment*, *22*(6), e2750.

Rowcliffe, M. (2023). activity: Animal Activity Statistics. R package version 1.3.4, <https://CRAN.R-project.org/package=activity>.

Sanders, S., & Kirschbaum, J. (2023). Woody species response to altered herbivore pressure at Isle Royale National Park. *Ecosphere*, *14*(7), e4623.

Sappington, J. M., Longshore, K. M., & Thompson, D. B. (2007). Quantifying landscape ruggedness for animal habitat analysis: a case study using bighorn sheep in the Mojave Desert. *The Journal of wildlife management*, *71*(5), 1419-1426.

Shelton, N. (1975). The Life of Isle Royale. Office of Publications, National Park Service, U.S. Department of the Interior, Washington.

Shiratsuru, S., & Pauli, J. N. (2024). Food-safety trade-offs drive dynamic behavioural antipredator responses among snowshoe hares. *Journal of Animal Ecology*, *93*(11), 1710-1721.

Sievert, P. R., & Keith, L. B. (1985). Survival of snowshoe hares at a geographic range boundary. *The Journal of wildlife management*, 854-866.

Signer, J., Fieberg, J., & Avgar, T. (2019). Animal movement tools (amt): R package for managing tracking data and conducting habitat selection analyses. *Ecology and evolution*, *9*(2), 880-890.

Studd, E. K., Boudreau, M. R., Majchrzak, Y. N., Menzies, A. K., Peers, M. J., Seguin, J. L., Lavergne, S. G., Boonstra, R., Murray, D. L., Boutin, S., & Humphries, M. M. (2019). Use of acceleration and acoustics to classify behavior, generate time budgets, and evaluate responses to moonlight in free-ranging snowshoe hares. Frontiers in Ecology and Evolution, 7, 154

Therneau T (2023). A Package for Survival Analysis in R. R package version 3.5-7, <https://CRAN.R-project.org/package=survival>.

United States Geological Survey (2021). United States Geological Survey 3D Elevation Program 1 arc-second Digital Elevation Model. Distributed by OpenTopography. https://doi.org/10.5069/G98K778D. Accessed: 2025-01-21.

USDA Forest Service. (2023). USFS NLCD Percent Tree Canopy CONUS v2021-4. Sioux Falls, SD.

Vazquez, C., Rowcliffe, J. M., Spoelstra, K., & Jansen, P. A. (2019). Comparing diel activity patterns of wildlife across latitudes and seasons: Time transformations using day length. *Methods in Ecology and Evolution*, *10*(12), 2057-2066.

Wilson, E. C., Zuckerberg, B., Peery, M. Z., & Pauli, J. N. (2022). Experimental repatriation of snowshoe hares along a southern range boundary reveals historical community interactions. *Ecological Monographs*, *92*(3), e1509.

Wisconsin Department of Natural Resources. (2016). Wiscland 2 land cover user guide. https://dnr.wisconsin.gov/maps/WISCLAND

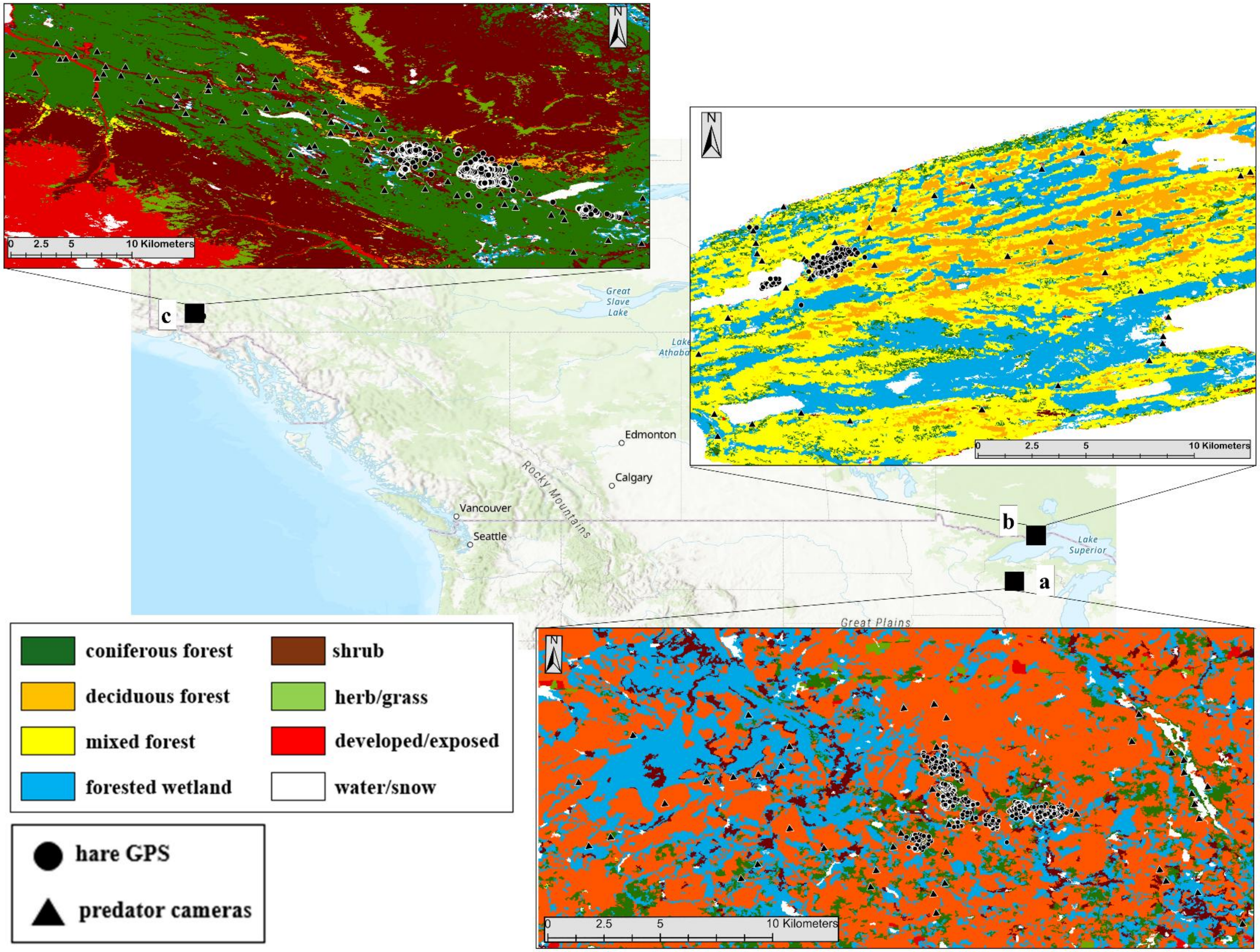

Figure S1. Map of the monitoring areas for snowshoe hares *Lepus americanus* and predators in (a) Wisconsin (cover types from Wiscland 2; Wisconsin Department of Natural Resources 2016), (b) Isle Royale (cover types from National land cover database (NLCD) 2019; Dewitz 2021) and (c) Yukon (cover types from Land cover 2019 Version 2; Hermosilla et al. 2022). Black circles represent the GPS locations of the hares (Wisconsin: winter 2022-2023, Isle Royale: winter 2022-2024, Yukon: winter 2018-2019) and black triangles represent the locations of the predator detection cameras (Wisconsin: winter 2022-2023, Isle Royale: winter 2021-2022, Yukon: winter 2018-2019).

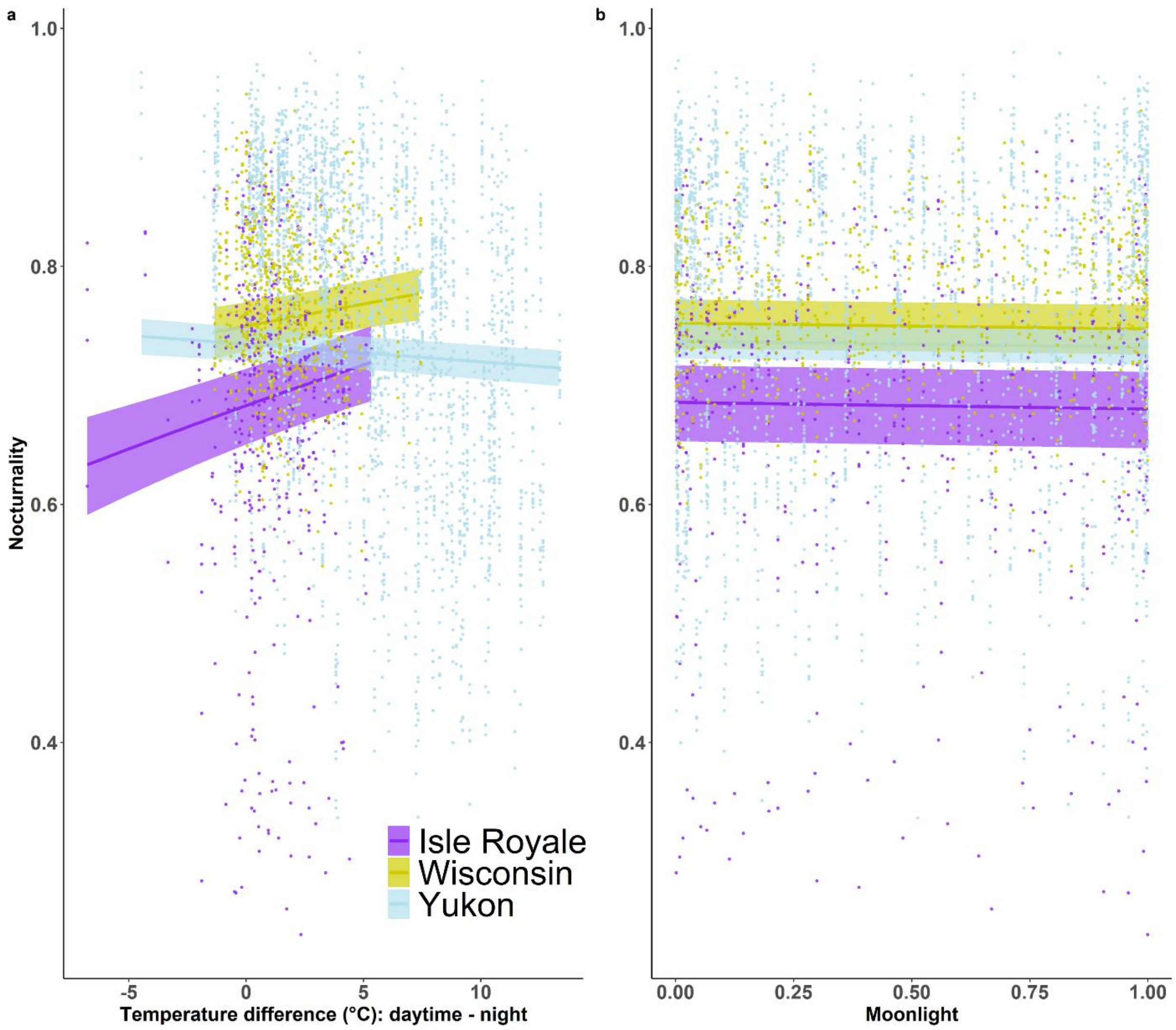

Figure S2. Effects of (a) temperature difference between daytime and night (on Julian day = 68 when night length is equal across the sites and fraction of the moon illuminated = 0.5) and (b) moonlight (on Julian day = 68 and day-night temperature difference = 0) on snowshoe hare *Lepus americanus* nocturnality (proportion of night activity time over daily activity time for each hare for each day) predicted by the generalized linear mixed effects model, presented with 95% confidence intervals. Small dots represent raw data points.

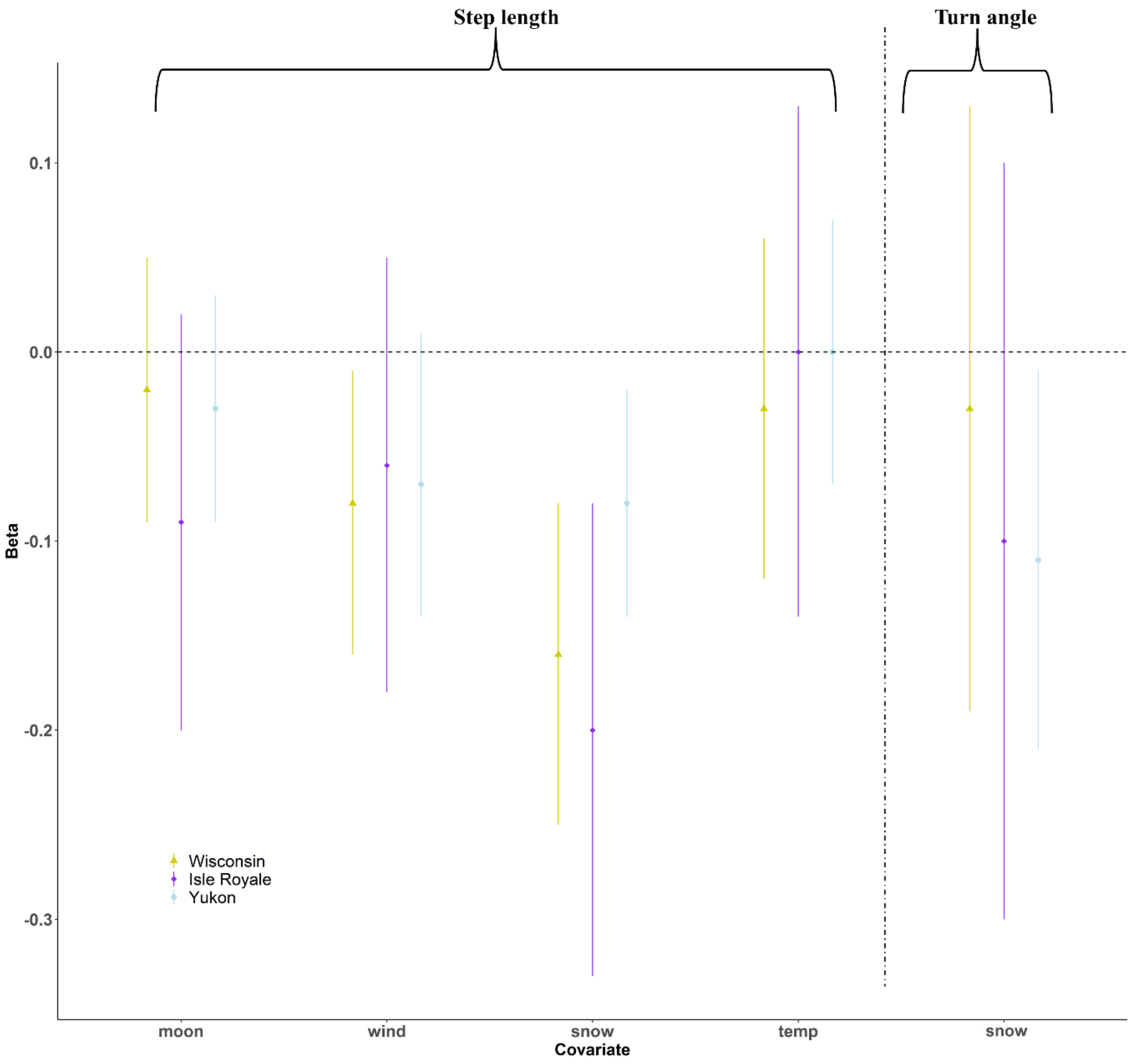

Figure S3. Effects of environmental covariates on snowshoe hare *Lepus americanus* night movement (for step length and cosine of turn angle) predicted by top integrated step selection analysis [iSSA] models in (a) Wisconsin, (b) Isle Royale and (c) Yukon. Snow is daily snow depth, wind is wind speed, temp is temperature and moon is the fraction of moon illuminated. All the environmental covariates were standardized in hare iSSA models, and error bars represent 95% confidence intervals.

Table S1. List of the landscape variables used in the integrated step selection models of snowshoe hares *Lepus americanus* and single-species occupancy models of predator species for Wisconsin, Isle Royale and Yukon, with spatial resolution and data sources.

Variable	Site	Resolution	Source
Cover type	Wisconsin	30 m	Wiscland 2 (Wisconsin Department of Natural Resources 2016)
	Isle Royale	30 m	National land cover database (NLCD) 2019 (Dewitz 2021)
	Yukon	30 m	Land cover 2019 Version 2 (Hermosilla et al. 2022)
Canopy cover	Wisconsin	30 m	USFS NLCD Percent Tree Canopy CONUS v2021-4 (USDA Forest Service 2023)
	Isle Royale	30 m	USFS NLCD Percent Tree Canopy CONUS v2021-4 (USDA Forest Service 2023)
	Yukon	30 m	Forest Percent Above Mean 2015 (Matasci et al. 2018)
Tree density	Wisconsin	30 m	TreeMap 2016 (Riley et al. 2022)
	Isle Royale	30 m	TreeMap 2016 (Riley et al. 2022)
	Yukon	1:5000 polygon	Vegetation Inventory (Government of Yukon 2025)
Elevation	Wisconsin	1 arc-second (30 m)	Digital Elevation Model (USGS 2021)
	Isle Royale	1 arc-second	Digital Elevation Model (USGS 2021)
	Yukon	0.75 arc-second	Canadian Digital Elevation Model (Natural Resources Canada 2013)

Table S2. Number of predator detections by trail cameras in winter (January-April) in Wisconsin (2022-2023), Isle Royale (2021-2022), and Yukon (2018-2019).

**Wisconsin**		
	2022 (457 camera-weeks)	2023 (255 camera-weeks)
**Canids**	20	23
**Mustelids**	36	15
Coyote	15	21
Red fox	3	1
Coyote/fox	2	1
Fisher	30	15
Ermine	6	0
**Isle Royale**		
	2021 (675 camera-weeks)	2022 (620 camera-weeks)
**Red fox**	255	139
**Yukon**		
	2018 (1163 camera-weeks)	2019 (1113 camera-weeks)
**Canada lynx**	213	133
**Coyote**	29	67

Table S3. Comparison of candidate generalized linear mixed effects models with beta distribution predicting daily nocturnality (calculated as the proportion of nighttime activity time over the daily [24h] activity time for each individual for each day) of snowshoe hares *Lepus americanus* in Wisconsin, Isle Royale and Yukon and parameter estimates from the selected model. Temperature is the difference in temperature between daytime and nighttime on the same day (positive values indicate that daytime was warmer than nighttime), and moon is the fraction of the moon illuminated. CIs are 95% confidence intervals.

Model	ΔAICc	AICc weight
Julian day*site + temperature*site + moon + (1|hare)	0.00	0.757
Julian day*site + temperature*site + moon*site + (1|hare)	2.27	0.243
Julian day*site + temperature + moon + (1|hare)	46.43	0.000
Julian day*site + temperature + moon*site + (1|hare)	47.80	0.000
Julian day + temperature*site + moon + (1|hare)	355.09	0.000
Julian day + temperature*site + moon*site + (1|hare)	356.45	0.000
Julian day + temperature + moon + site + (1|hare)	458.17	0.000
Julian day + temperature + moon*site + (1|hare)	459.95	0.000

Fixed effect	ß	lower CI	upper CI	SE	z value
Intercept	1.58	1.45	1.70	0.06	24.61
Julian day	-0.01	-0.01	-0.01	0.00	-11.04
Site: Isle Royale	0.11	-0.10	0.32	0.11	1.05
Site: Yukon	0.80	0.65	0.95	0.07	10.78
Temperature	0.02	0.01	0.03	0.01	3.30
Moon	-0.03	-0.05	0.00	0.01	-2.01
Julian day: Isle Royale	-0.01	-0.01	0.00	0.00	-7.09
Julian day: Yukon	-0.01	-0.01	-0.01	0.00	-18.97
Temperature: Isle Royale	0.01	-0.01	0.03	0.01	1.37
Temperature: Yukon	-0.03	-0.04	-0.02	0.01	-4.35

Random effect	Variance	SD
Hare ID	0.08	0.28

Table S4. Comparison of candidate integrated step selection models and parameter estimates from the selected model for night steps of snowshoe hares *Lepus americanus* in Wisconsin. %conifer is percent coniferous forests, %deciduous is percent deciduous forests, tree is tree density, VRM is vector ruggedness measurement, snow is daily snow depth and moon is the fraction of moon illuminated. All the candidate models have the same structure for movement: [Movement] = log(SL) + log(SL):moon + log(SL):snow + log(SL):wind + log(SL):temperature + cos(TA) + cos(TA):snow, where SL is step length, TA is turn angle and wind is wind speed. Akaike information criterion for small sample size (AICc) model weight is presented as the mean value of the 1000 bootstrap iterations for each model and % top is the percent of each model being selected as the top model over the 1000 iterations. All the landscape and environmental covariates were standardized, and CIs are 95% bootstrap confidence intervals.

**Model**	**AICc weight**	**% top**
%conifer + %deciduous + canopy + tree + elevation + VRM + %conifer:snow + %deciduous:tree + [Movement]	0.106	19.1
%conifer + %deciduous + canopy + tree + elevation + VRM + %conifer:snow + %conifer:tree + [Movement]	0.070	10.2
%conifer + %deciduous + canopy + tree + elevation + VRM + %conifer:moon + %deciduous:tree + [Movement]	0.064	9
%conifer + %deciduous + canopy + tree + elevation + VRM + %conifer:snow + %conifer:moon + [Movement]	0.057	7.7
%conifer + %deciduous + canopy + tree + elevation + VRM + %deciduous:tree + [Movement]	0.047	7.1
%conifer + %deciduous + canopy + tree + elevation + VRM + %conifer:moon + %conifer:tree [Movement]	0.042	4.8
%conifer + %deciduous + canopy + tree + elevation + VRM + %conifer:snow + %deciduous:moon + [Movement]	0.040	3.9
%conifer + %deciduous + canopy + tree + elevation + VRM + %deciduous:moon + %deciduous:tree + [Movement]	0.039	3.2
%conifer + %deciduous + canopy + tree + elevation + VRM + %conifer:snow + [Movement]	0.038	4.4
%conifer + %deciduous + canopy + tree + elevation + VRM + %conifer:tree + [Movement]	0.036	4.5
%conifer + %deciduous + canopy + tree + elevation + VRM + %conifer:tree + %deciduous:tree + [Movement]	0.035	0.9
%conifer + %deciduous + canopy + tree + elevation + VRM + %conifer:tree + %deciduous:moon + [Movement]	0.032	2.7
%conifer + %deciduous + canopy + tree + elevation + VRM + canopy:moon + %deciduous:tree + [Movement]	0.031	2
%conifer + %deciduous + canopy + tree + elevation + VRM + %deciduous:snow + %deciduous:tree + [Movement]	0.030	1.1
%conifer + %deciduous + canopy + tree + elevation + VRM + tree:snow + %deciduous:tree + [Movement]	0.028	1.6
%conifer + %deciduous + canopy + tree + elevation + VRM + %conifer:snow + canopy:moon + [Movement]	0.026	1.7
%conifer + %deciduous + canopy + tree + elevation + VRM + %conifer:tree + %deciduous:snow + [Movement]	0.025	1.7
%conifer + %deciduous + canopy + tree + elevation + VRM + %conifer:snow + %deciduous:snow + [Movement]	0.024	1.5
%conifer + %deciduous + canopy + tree + elevation + VRM + %conifer:tree + canopy:moon + [Movement]	0.023	1.7

%conifer + %deciduous + canopy + tree + elevation + VRM + %conifer:snow + tree:snow + [Movement]	0.022	1
%conifer + %deciduous + canopy + tree + elevation + VRM + %conifer:tree + tree:snow + [Movement]	0.020	0.8
%conifer + %deciduous + canopy + tree + elevation + VRM + %conifer:moon + [Movement]	0.018	1.1
%conifer + %deciduous + canopy + tree + elevation + VRM + %conifer:moon + %deciduous:snow + [Movement]	0.014	0.9
%conifer + %deciduous + canopy + tree + elevation + VRM + [Movement]	0.014	2.5
%conifer + %deciduous + canopy + tree + elevation + VRM + %conifer:moon + %deciduous:moon + [Movement]	0.014	1.1
%conifer + %deciduous + canopy + tree + elevation + VRM + %deciduous:moon + [Movement]	0.013	0.6
%conifer + %deciduous + canopy + tree + elevation + VRM + %conifer:moon + canopy:moon + [Movement]	0.012	0.6
%conifer + %deciduous + canopy + tree + elevation + VRM + %deciduous:snow + [Movement]	0.010	0.7
%conifer + %deciduous + canopy + tree + elevation + VRM + %deciduous:snow + %deciduous:moon + [Movement]	0.010	0.1
%conifer + %deciduous + canopy + tree + elevation + VRM + canopy:moon + %deciduous:moon + [Movement]	0.009	0.6
%conifer + %deciduous + canopy + tree + elevation + VRM + %conifer:moon + tree:snow + [Movement]	0.009	0.2
%conifer + %deciduous + canopy + tree + elevation + VRM + canopy:moon + [Movement]	0.009	0.3
%conifer + %deciduous + canopy + tree + elevation + VRM + tree:snow + [Movement]	0.007	0.5
%conifer + %deciduous + canopy + tree + elevation + VRM + tree:snow + %deciduous:moon + [Movement]	0.007	0.2
%conifer + %deciduous + canopy + tree + elevation + VRM + canopy:moon + %deciduous:snow + [Movement]	0.007	0
%conifer + %deciduous + canopy + tree + elevation + VRM + tree:snow + %deciduous:snow + [Movement]	0.006	0
%conifer + %deciduous + canopy + tree + elevation + VRM + canopy:moon + tree:snow + [Movement]	0.005	0

Variable	Mean	Lower CI	Upper CI
%conifer	0.03	-0.13	0.20
%deciduous	-0.03	-0.19	0.15
tree	-0.16	-0.33	-0.01
%conifer:snow	0.12	-0.01	0.24
%deciduous:tree	-0.13	-0.26	-0.01
canopy	-0.01	-0.14	0.13
elevation	0.07	-0.20	0.34
VRM	-0.12	-0.26	0.00
log(SL)	0.04	-0.03	0.13
cos(TA)	-0.56	-0.78	-0.36
log(SL):moon	-0.02	-0.09	0.05
log(SL):snow	-0.16	-0.25	-0.08

log(SL):wind	-0.08	-0.16	-0.01
log(SL):temperature	-0.03	-0.12	0.06
cos(TA):snow	-0.03	-0.19	0.13

Table S5. Comparison of candidate integrated step selection models and parameter estimates from the selected model for daytime steps of snowshoe hares *Lepus americanus* in Wisconsin. %conifer is percent coniferous forests, %deciduous is percent deciduous forests, tree is tree density, VRM is vector ruggedness measurement, and snow is daily snow depth. All the candidate models have the same structure for movement: [Movement] = log(SL) + log(SL):snow + log(SL):wind + log(SL):temperature + cos(TA) + cos(TA):snow, where SL is step length, TA is turn angle and wind is wind speed. Akaike information criterion for small sample size (AICc) model weight is presented as the mean value of the 1000 bootstrap iterations for each model and % top is the percent of each model being selected as the top model over the 1000 iterations. All the landscape and environmental covariates were standardized, and CIs are 95% bootstrap confidence intervals.

**Model**	**AICc weight**	**% top**
%conifer + %deciduous + canopy + tree + elevation + VRM + [Movement]	0.138	46.9
%conifer + %deciduous + canopy + tree + elevation + VRM + %conifer:snow + [Movement]	0.109	17.4
%conifer + %deciduous + canopy + tree + elevation + VRM + %conifer:tree + [Movement]	0.075	6.5
%conifer + %deciduous + canopy + tree + elevation + VRM + %deciduous:tree + [Movement]	0.073	5.8
%conifer + %deciduous + canopy + tree + elevation + VRM + tree:snow + [Movement]	0.072	5.6
%conifer + %deciduous + canopy + tree + elevation + VRM + %deciduous:snow + [Movement]	0.070	3.7
%conifer + %deciduous + canopy + tree + elevation + VRM + %conifer:snow + %deciduous:snow + [Movement]	0.064	4.4
%conifer + %deciduous + canopy + tree + elevation + VRM + %conifer:snow + tree:snow + [Movement]	0.056	2.2
%conifer + %deciduous + canopy + tree + elevation + VRM + %conifer:snow + %conifer:tree + [Movement]	0.056	1.5
%conifer + %deciduous + canopy + tree + elevation + VRM + %conifer:snow + %deciduous:tree + [Movement]	0.055	1.7
%conifer + %deciduous + canopy + tree + elevation + VRM + %conifer:tree + %deciduous:tree + [Movement]	0.040	1.1
%conifer + %deciduous + canopy + tree + elevation + VRM + tree:snow + %deciduous:tree + [Movement]	0.039	0.6
%conifer + %deciduous + canopy + tree + elevation + VRM + %conifer:tree + %deciduous:snow + [Movement]	0.039	0.9
%conifer + %deciduous + canopy + tree + elevation + VRM + %conifer:tree + tree:snow + [Movement]	0.039	0.6
%conifer + %deciduous + canopy + tree + elevation + VRM + %deciduous:snow + %deciduous:tree + [Movement]	0.037	0.7
%conifer + %deciduous + canopy + tree + elevation + VRM + tree:snow + %deciduous:snow + [Movement]	0.037	0.4

**Variable**	**Mean**	**Lower CI**	**Upper CI**
%conifer	0.21	-0.06	0.48
%deciduous	0.03	-0.26	0.31
tree	-0.17	-0.47	0.09
canopy	-0.10	-0.31	0.13
elevation	-0.18	-0.82	0.46

VRM	-0.20	-0.44	0.00
log(SL)	0.01	-0.05	0.07
cos(TA)	-0.56	-0.84	-0.31
log(SL):snow	-0.05	-0.12	0.01
log(SL):wind	-0.06	-0.12	0.00
log(SL):temperature	-0.06	-0.14	0.00
cos(TA):snow	-0.04	-0.27	0.17

Table S6. Comparison of single-species occupancy models and parameter estimates from the selected models for canid and mustelid predators in Wisconsin. %conifer is percent coniferous forests, %deciduous is percent deciduous forests, tree is tree density, canopy is percent canopy cover, VRM is vector ruggedness measurement, dist_road is distance to the nearest road, temperature is weekly average of daily mean temperature, wind is weekly average of daily mean wind speed, snow is weekly average of daily snow depth and moon is weekly average of fraction of the moon illuminated. The degrees of freedom (df), difference in Akaike information criterion for small sample size (AICc) values (ΔAIC), and AICc model weight are provided. Note that the model with landscape variables as occupancy covariates is not shown due to its singular fit for both canid and mustelid predators. *p* represents detection probability and *ψ* represents occupancy probability. All the landscape and environmental covariates were standardized, and CIs are 95% confidence intervals.

Canid

Model	df	ΔAICc	AICc weight
p(%conifer*snow + %deciduous*tree + canopy + elevation + VRM + dist_road + temperature + snow + year + (1|site)), *ψ*(·)	15	0	0.762
p(%conifer*snow + %deciduous*tree + canopy + elevation + VRM + dist_road + temperature + snow + year + (1|site)), *ψ*(year)	16	2.33	0.238

Detection

Fixed effect	Estimate	Lower CI	Upper CI	SE	*z*-value
Intercept	-3.23	-4.29	-2.18	0.54	-6.00
%conifer	-0.61	-1.43	0.22	0.42	-1.44
%conifer:snow	-0.55	-1.07	-0.03	0.26	-2.09
%deciduous	-0.14	-1.10	0.82	0.49	-0.28
tree	0.54	-0.48	1.55	0.52	1.03
%deciduous:tree	-0.27	-1.09	0.56	0.42	-0.63
canopy	0.11	-0.55	0.77	0.34	0.33
elevation	1.00	0.38	1.62	0.32	3.17
VRM	0.24	-0.16	0.64	0.20	1.20
dist_road	0.09	-0.41	0.60	0.26	0.36
temperature	0.22	-0.49	0.93	0.36	0.60
snow	-0.59	-1.28	0.10	0.35	-1.67
year:2023	1.78	0.46	3.10	0.67	2.64

Random effect	Variance	SD
site	0	0.003

Occupancy (logit scale)

Estimate	SE	*z*-value
0.598	0.539	1.11

MacKenzie and Bailey Goodness-of-fit Test

c-hat	P
0.70	0.67

Mustelid

Model	Df	ΔAICc	AICc weight
p(%conifer*snow + %deciduous*tree + canopy + elevation + VRM + dist_road + temperature + snow + year + (1|site)), *ψ*(year)	16	0	0.771
p(%conifer*snow + %deciduous*tree + canopy + elevation + VRM + dist_road + temperature + snow + year + (1|site)), *ψ*(·)	15	2.45	0.227
p(%conifer*snow + %deciduous*tree + canopy + elevation + VRM + dist_road + temperature + snow + year + (1|site)), *ψ*(%conifer + % deciduous + tree + canopy + elevation + VRM + dist_road)	23	11.12	0.003

Detection

Fixed effect	Estimate	Lower CI	Upper CI	adjusted SE	*z*-value
Intercept	-2.45	-3.16	-1.75	0.43	-6.42
%conifer	0.19	-0.32	0.70	0.31	0.67
%conifer:snow	0.04	-0.30	0.37	0.20	0.19
%deciduous	-0.90	-1.77	-0.03	0.53	-1.90
tree	0.28	-0.44	1.01	0.44	0.71
%deciduous:tree	0.13	-0.56	0.82	0.42	0.34
canopy	0.12	-0.36	0.60	0.29	0.45
elevation	0.36	-0.10	0.82	0.28	1.44
VRM	0.09	-0.26	0.43	0.21	0.46
dist_road	0.06	-0.34	0.45	0.24	0.26
temperature	-0.06	-0.64	0.52	0.35	-0.19
snow	-0.05	-0.66	0.56	0.37	-0.15
year:2023	1.10	-0.30	2.50	0.85	1.44

Random effect	Variance	SD
site	0.372	0.61

Occupancy (logit scale)

	Estimate	adjustedSE	*z*-value
Intercept	11.7	178.9	0.073
year:2023	-12.1	178.9	-0.076

MacKenzie and Bailey Goodness-of-fit Test

c-hat	P
1.24	0.15

Table S7. Comparison of candidate integrated step selection models and parameter estimates from the selected model for night steps of snowshoe hares *Lepus americanus* in Isle Royale. %mix is percent mixed forests, %wet is percent forested wetlands, percent, tree is tree density, VRM is vector ruggedness measurement, snow is daily snow depth and moon is the fraction of moon illuminated. All the candidate models have the same structure for movement: [Movement] = log(SL) + log(SL):moon + log(SL):snow + log(SL):wind + log(SL):temperature + cos(TA) + cos(TA):snow, where SL is step length, TA is turn angle and wind is wind speed. Akaike information criterion for small sample size (AICc) model weight is presented as the mean value of the 1000 bootstrap iterations for each model and % top is the percent of each model being selected as the top model over the 1000 iterations. All the landscape and environmental covariates were standardized, and CIs are bootstrap 95% confidence intervals.

**Model**	**AICc weight**	**% top**
%mix + canopy + tree + elevation + VRM + %mix:tree + [Movement]	0.121	32.2
%mix + canopy + tree + elevation + VRM + %mix:tree + canopy:moon + [Movement]	0.081	7.5
%mix + canopy + tree + elevation + VRM + %mix:tree + tree:snow + [Movement]	0.077	7.9
%mix + canopy + tree + elevation + VRM + %mix:moon + %mix:tree + [Movement]	0.067	4.3
%mix + canopy + tree + elevation + VRM + %mix:snow + %mix:tree + [Movement]	0.062	2.9
%mix + canopy + tree + elevation + VRM + [Movement]	0.059	14.8
%mix + canopy + tree + elevation + VRM + canopy:moon + [Movement]	0.041	4.4
%mix + canopy + tree + elevation + VRM + tree:snow + [Movement]	0.036	3.1
%mix + canopy + tree + elevation + VRM + %mix:moon + [Movement]	0.035	2.4
%mix + canopy + tree + elevation + VRM + %mix:snow + [Movement]	0.031	2.3
%wet + canopy + tree + elevation + VRM + [Movement]	0.030	3.8
%wet + canopy + tree + elevation + VRM + %wet:tree + [Movement]	0.027	2.2
%mix + canopy + tree + elevation + VRM + canopy:moon + tree:snow + [Movement]	0.025	1.3
%mix + canopy + tree + elevation + VRM + %mix:moon + canopy:moon + [Movement]	0.022	0.5
%mix + canopy + tree + elevation + VRM + %mix:snow + canopy:moon + [Movement]	0.021	0.6
%mix + canopy + tree + elevation + VRM + %mix:moon + tree:snow + [Movement]	0.021	0.6
%wet + canopy + tree + elevation + VRM + canopy:moon + [Movement]	0.020	1
%wet + canopy + tree + elevation + VRM + %wet:moon + %wet:tree + [Movement]	0.020	1.6
%wet + canopy + tree + elevation + VRM + %wet:moon + [Movement]	0.019	1.3
%wet + canopy + tree + elevation + VRM + tree:snow + [Movement]	0.019	0.9
%mix + canopy + tree + elevation + VRM + %mix:snow + tree:snow + [Movement]	0.018	0.1
%mix + canopy + tree + elevation + VRM + %mix:snow + %mix:moon + [Movement]	0.018	0.4
%wet + canopy + tree + elevation + VRM + %wet:tree + canopy:moon + [Movement]	0.018	0.7

%wet + canopy + tree + elevation + VRM + %wet:tree + tree:snow + [Movement]	0.016	0.4
%wet + canopy + tree + elevation + VRM + %wet:snow + [Movement]	0.016	0.8
%wet + canopy + tree + elevation + VRM + %wet:moon + canopy:moon + [Movement]	0.014	0.9
%wet + canopy + tree + elevation + VRM + %wet:snow + %wet:tree + [Movement]	0.014	0.3
%wet + canopy + tree + elevation + VRM + canopy:moon + tree:snow + [Movement]	0.012	0
%wet + canopy + tree + elevation + VRM + %wet:moon + tree:snow + [Movement]	0.012	0.1
%wet + canopy + tree + elevation + VRM + %wet:snow + canopy:moon + [Movement]	0.011	0.3
%wet + canopy + tree + elevation + VRM + %wet:snow + %wet:moon + [Movement]	0.011	0.3
%wet + canopy + tree + elevation + VRM + %wet:snow + tree:snow + [Movement]	0.009	0.1

**Variable**	**Mean**	**Lower CI**	**Upper CI**
%mix	-0.19	-0.33	-0.04
tree	0.27	0.11	0.43
%mix:tree	0.15	0.01	0.29
canopy	0.45	0.23	0.73
elevation	-0.33	-0.55	-0.13
VRM	-0.11	-0.46	0.11
log(SL)	0.06	-0.04	0.18
cos(TA)	-0.45	-0.72	-0.21
log(SL):moon	-0.09	-0.20	0.02
log(SL):snow	-0.20	-0.33	-0.08
log(SL):wind	-0.06	-0.18	0.05
log(SL):temperature	0.00	-0.14	0.13
cos(TA):snow	-0.10	-0.30	0.10

Table S8. Comparison of candidate integrated step selection models and parameter estimates from the selected model for daytime steps of snowshoe hares *Lepus americanus* in Isle Royale. %mix is percent mixed forests, %wet is percent forested wetlands, percent, tree is tree density, VRM is vector ruggedness measurement, and snow is daily snow depth. All the candidate models have the same structure for movement: [Movement] = log(SL) + log(SL):snow + log(SL):wind + log(SL):temperature + cos(TA) + cos(TA):snow, where SL is step length, TA is turn angle and wind is wind speed. Akaike information criterion for small sample size (AICc) model weight is presented as the mean value of the 1000 bootstrap iterations for each model and % top is the percent of each model being selected as the top model over the 1000 iterations. All the landscape and environmental covariates were standardized, and CIs are bootstrap 95% confidence intervals.

Model	AICc weight	% top
%mix + canopy + tree + elevation + VRM + [Movement]	0.157	35.5
%wet + canopy + tree + elevation + VRM + [Movement]	0.141	28.4
%mix + canopy + tree + elevation + VRM + %mix:tree + [Movement]	0.094	10.2
%mix + canopy + tree + elevation + VRM + tree:snow + [Movement]	0.082	5.2
%mix + canopy + tree + elevation + VRM + %mix:snow + [Movement]	0.076	3.8
%wet + canopy + tree + elevation + VRM + %wet:snow + [Movement]	0.075	6
%wet + canopy + tree + elevation + VRM + tree:snow + [Movement]	0.073	4.3
%wet + canopy + tree + elevation + VRM + %wet:tree + [Movement]	0.067	2.7
%mix + canopy + tree + elevation + VRM + %mix:tree + tree:snow + [Movement]	0.047	1
%mix + canopy + tree + elevation + VRM + %mix:snow + %mix:tree + [Movement]	0.043	0.6
%wet + canopy + tree + elevation + VRM + %wet:snow + tree:snow + [Movement]	0.041	1.3
%mix + canopy + tree + elevation + VRM + %mix:snow + tree:snow + [Movement]	0.037	0.3
%wet + canopy + tree + elevation + VRM + %wet:snow + %wet:tree + [Movement]	0.034	0.4
%wet + canopy + tree + elevation + VRM + %wet:tree + tree:snow + [Movement]	0.034	0.3

Variable	Mean	Lower CI	Upper CI
%mix	-0.14	-0.39	0.08
tree	-0.02	-0.21	0.18
canopy	0.15	-0.04	0.37
elevation	0.15	-0.36	0.62
VRM	-0.31	-0.80	0.04
log(SL)	0.06	-0.03	0.17
cos(TA)	-0.42	-0.76	-0.13
log(SL):snow	-0.02	-0.12	0.09
log(SL):wind	-0.05	-0.15	0.05
log(SL):temperature	-0.01	-0.12	0.10
cos(TA):snow	0.04	-0.24	0.35

Table S9. Comparison of single-species occupancy models and parameter estimates from the selected model for red foxes *Vulpes vulpes* in Isle Royale. %mix is percent mixed forests, tree is tree density, VRM is vector ruggedness measurement, temperature is weekly average of daily mean temperature, wind is weekly average of daily mean wind speed, snow is weekly average of daily snow depth and moon is weekly average of fraction of the moon illuminated. The degrees of freedom (df), difference in Akaike information criterion for small sample size (AICc) values (ΔAIC), and AICc model weight are provided. *p* represents detection probability and *ψ* represents occupancy probability. All the landscape and environmental covariates were standardized, and CIs are 95% confidence intervals.

Model	df	ΔAICc	AICc weight
p(%mix*tree + canopy + elevation + VRM + temperature + snow + year + (1|site)), *ψ*(·)	12	0	0.725
p(%mix*tree + canopy + elevation + VRM + temperature + snow + year + (1|site)), *ψ*(year)	13	1.94	0.275

Detection

Fixed effect	Estimate	Lower CI	Upper CI	SE	*z*-value
Intercept	-0.73	-1.32	-0.14	0.30	-2.41
%mix	0.08	-0.34	0.50	0.22	0.37
tree	0.16	-0.30	0.62	0.23	0.70
%mix:tree	0.09	-0.25	0.42	0.17	0.50
canopy	-0.19	-0.59	0.21	0.20	-0.95
elevation	-0.13	-0.48	0.21	0.18	-0.75
VRM	-0.02	-0.35	0.31	0.17	-0.14
temperature	0.57	0.34	0.80	0.12	4.94
snow	0.27	-0.23	0.76	0.25	1.05
year:2022	-0.79	-1.80	0.22	0.51	-1.54

Random effect	Variance	SD
site	0.471	0.69

Occupancy (logit scale)

Estimate	SE	*z*-value
3.55	0.898	3.96

MacKenzie and Bailey Goodness-of-fit Test

c-hat	P
0.92	0.49

Table S10. Comparison of candidate integrated step selection models and parameter estimates from the selected model for night steps of snowshoe hares *Lepus americanus* in Yukon. %conifer is percent coniferous forests, %shrub is percent shrub, tree is tree density, VRM is vector ruggedness Measurement, snow is daily snow depth and moon is the fraction of moon illuminated. All the candidate models have the same structure for movement: [Movement] = log(SL) + log(SL):moon + log(SL):snow + log(SL):wind + log(SL):temperature + cos(TA) + cos(TA):snow, where SL is step length, TA is turn angle and wind is wind speed. Akaike information criterion for small sample size (AICc) model weight is presented as the mean value of the 1000 bootstrap iterations for each model and % top is the percent of each model being selected as the top model over the 1000 iterations. All the landscape and environmental covariates were standardized, and CIs are bootstrap 95% confidence intervals.

**Model**	**AICc weight**	**% top**
%conifer + tree + elevation + VRM + [Movement]	0.158	44.9
%conifer + tree + elevation + VRM + %conifer:moon + [Movement]	0.113	13.2
%conifer + tree + elevation + VRM + %conifer:tree + [Movement]	0.111	11.5
%conifer + tree + elevation + VRM + tree:snow + [Movement]	0.093	7.1
%conifer + tree + elevation + VRM + %conifer:snow + [Movement]	0.090	6.1
%conifer + tree + elevation + VRM + %conifer:moon + %conifer:tree + [Movement]	0.082	3.5
%conifer + tree + elevation + VRM + %conifer:tree + tree:snow + [Movement]	0.066	2.5
%conifer + tree + elevation + VRM +%conifer:moon + tree:snow + [Movement]	0.065	1.6
%conifer + tree + elevation + VRM + %conifer:snow + %conifer:tree + [Movement]	0.064	2.3
%conifer + tree + elevation + VRM + %conifer:snow + %conifer:moon + [Movement]	0.064	1.8
%conifer + tree + elevation + VRM + %conifer:snow + tree:snow + [Movement]	0.058	2.6
%shrub + elevation + VRM + [Movement]	0.011	1.3
%shrub + elevation + VRM + %shrub:snow + [Movement]	0.009	1
%shrub + elevation + VRM + %shrub:moon + [Movement]	0.009	0.5
%shrub + canopy + tree + elevation + VRM + %shrub:moon + %shrub:snow + [Movement]	0.007	0.1

**Variable**	**Mean**	**Lower CI**	**Upper CI**
%conifer	0.14	0.05	0.23
tree	0.15	0.04	0.25
elevation	-0.05	-0.26	0.16
VRM	0.09	0.02	0.16
log(SL)	0.05	-0.01	0.11
cos(TA)	-0.51	-0.64	-0.38
log(SL):moon	-0.03	-0.09	0.03
log(SL):snow	-0.08	-0.14	-0.02
log(SL):wind	-0.07	-0.14	0.01
log(SL):temperature	0.00	-0.07	0.07
cos(TA):snow	-0.11	-0.21	-0.01

Table S11. Comparison of candidate integrated step selection models and parameter estimates from the selected model for daytime steps of snowshoe hares *Lepus americanus* in Yukon. %conifer is percent coniferous forests, %shrub is percent shrub, tree is tree density, VRM is vector ruggedness Measurement, and snow is daily snow depth. All the candidate models have the same structure for movement: [Movement] = log(SL) + log(SL):snow + log(SL):wind + log(SL):temperature + cos(TA) + cos(TA):snow, where SL is step length, TA is turn angle and wind is wind speed. Akaike information criterion for small sample size (AICc) model weight is presented as the mean value of the 1000 bootstrap iterations for each model and % top is the percent of each model being selected as the top model over the 1000 iterations. All the landscape and environmental covariates were standardized, and CIs are bootstrap 95% confidence intervals.

**Model**	**AICc weight**	**% top**
%conifer + tree + elevation + VRM + [Movement]	0.246	59.5
%conifer + tree + elevation + VRM + %conifer:snow + [Movement]	0.151	10.9
%conifer + tree + elevation + VRM + tree:snow + [Movement]	0.150	9.9
%conifer + tree + elevation + VRM + %conifer:tree + [Movement]	0.148	9.8
%conifer + tree + elevation + VRM + %conifer:tree + tree:snow + [Movement]	0.095	3.1
%conifer + tree + elevation + VRM + %conifer:snow + %conifer:tree + [Movement]	0.095	3.5
%conifer + tree + elevation + VRM + %conifer:snow + tree:snow + [Movement]	0.089	2.4
%shrub + elevation + VRM + [Movement]	0.016	0.9
%shrub + elevation + VRM + %shrub:snow + [Movement]	0.010	0

**Variable**	**Mean**	**Lower CI**	**Upper CI**
%conifer	0.26	0.12	0.39
tree	-0.24	-0.40	-0.10
elevation	-0.29	-0.79	0.22
VRM	0.13	-0.01	0.26
log(SL)	-0.01	-0.05	0.04
cos(TA)	-0.33	-0.49	-0.18
log(SL):snow	-0.01	-0.05	0.03
log(SL):wind	-0.05	-0.09	0.00
log(SL):temperature	-0.03	-0.08	0.02
cos(TA):snow	-0.07	-0.19	0.05

Table S12. Comparison of single-species occupancy models and parameter estimates from the selected models for lynx *Lynx canadensis* and coyotes *Canis latrans* in Yukon. %conifer is percent coniferous forests, tree is tree density, VRM is vector ruggedness measurement, dist_road is distance to the nearest road, temperature is weekly average of daily mean temperature, wind is weekly average of daily mean wind speed, snow is weekly average of daily snow depth and moon is weekly average of fraction of the moon illuminated. The degrees of freedom (df), difference in Akaike information criterion for small sample size (AICc) values (ΔAIC), and AICc model weight are provided. *p* represents detection probability and *ψ* represents occupancy probability. All the landscape and environmental covariates were standardized, and CIs are 95% confidence intervals.

Lynx

Model	df	ΔAICc	AICc weight
p(%conifer + tree + elevation + VRM + dist_road + temperature + snow + year + (1|site)), ψ(·)	11	0	0.708
p(%conifer + tree + elevation + VRM + dist_road + temperature + snow + year + (1|site)), ψ(year)	12	2.15	0.242
p(%conifer + tree + elevation + VRM + dist_road + temperature + snow + year + (1|site)), ψ(%conifer + tree + elevation + VRM + dist_road)	17	5.30	0.050

Detection

Fixed effect	Estimate	Lower CI	Upper CI	adjusted SE	*z*-value
Intercept	-0.89	-1.25	-0.54	0.22	-4.75
%conifer	0.01	-0.26	0.28	0.16	0.09
tree	-0.02	-0.29	0.25	0.16	-0.13
elevation	0.28	0.00	0.56	0.17	1.88
VRM	0.20	-0.02	0.41	0.13	1.76
dist_road	0.00	-0.27	0.27	0.17	0.00
temperature	0.27	0.08	0.46	0.11	2.74
snow	-0.27	-0.50	-0.03	0.14	-2.16
year:2019	-0.79	-1.31	-0.27	0.32	-2.87

Random effect	Variance	SD
site	0.382	0.618

Occupancy (logit scale)

Estimate	adjusted SE	*z*-value
1.16	0.34	3.87

MacKenzie and Bailey Goodness-of-fit Test

c-hat	P
1.33	0.14

Coyote

Model	df	ΔAICc	AICc weight

p(%conifer + tree + elevation + VRM + dist_road + temperature + snow + year + (1|site)), ψ(year)	12	0	0.764
p(%conifer + tree + elevation + VRM + dist_road + temperature + snow + year + (1|site)), ψ(·)	11	2.42	0.228
p(%conifer + tree + elevation + VRM + dist_road + temperature + snow + year + (1|site)), ψ(%conifer + tree + elevation + VRM + dist_road)	17	9.22	0.008

Detection

Fixed effect	Estimate	Lower CI	Upper CI	SE	*z*-value
Intercept	-2.00	-2.88	-1.11	0.45	-4.41
%conifer	-0.06	-0.45	0.32	0.20	-0.32
tree	0.21	-0.16	0.57	0.18	1.11
elevation	-0.65	-1.00	-0.29	0.18	-3.59
VRM	0.18	-0.12	0.48	0.15	1.18
dist_road	-0.06	-0.33	0.21	0.14	-0.41
temperature	0.45	0.10	0.80	0.18	2.54
snow	-0.31	-0.80	0.18	0.25	-1.23
year:2019	-0.51	-1.65	0.63	0.58	-0.88

Random effect	Variance	SD
site	0	0.001

Occupancy (logit scale)

	Estimate	SE	*z*-value
Intercept	-1.03	0.465	-2.21
year:2019	1.57	0.654	2.41

MacKenzie and Bailey Goodness-of-fit Test

c-hat	P
0.55	0.53
